# Independent Recruitment of Duplicated β-Subunit-Coding NAD-ME Genes Aided the Evolution of C4 Photosynthesis in Cleomaceae

**DOI:** 10.3389/fpls.2020.572080

**Published:** 2020-10-06

**Authors:** Marcos A. Tronconi, Meike Hüdig, M. Eric Schranz, Veronica G. Maurino

**Affiliations:** ^1^Centro de Estudios Fotosintéticos y Bioquímicos (CEFOBI-CONICET), Facultad de Ciencias Bioquímicas y Farmacéuticas, Universidad Nacional de Rosario, Rosario, Argentina; ^2^Abteilung Molekulare Pflanzenphysiologie, Institut für Molekulare Physiologie und Biotechnologie der Pflanzen, Rheinische Friedrich-Wilhelms-Universität Bonn, Bonn, Germany; ^3^Biosystematics Group, Wageningen University, Wageningen, Netherlands

**Keywords:** C_4_-photosynthesis, C_4_-evolution, Cleomaceae, gene duplication, NAD-malic enzyme, subfunctionalization, neofunctionalization

## Abstract

In different lineages of C_4_ plants, the release of CO_2_ by decarboxylation of a C_4_ acid near rubisco is catalyzed by NADP-malic enzyme (ME) or NAD-ME, and the facultative use of phosphoenolpyruvate carboxykinase. The co-option of gene lineages during the evolution of C_4_-NADP-ME has been thoroughly investigated, whereas that of C_4_-NAD-ME has received less attention. In this work, we aimed at elucidating the mechanism of recruitment of *NAD-ME* for its function in the C_4_ pathway by focusing on the eudicot family Cleomaceae. We identified a duplication of *NAD-ME* in vascular plants that generated the two paralogs lineages: α*-* and β*-NAD-ME*. Both gene lineages were retained across seed plants, and their fixation was likely driven by a degenerative process of sub-functionalization, which resulted in a NAD-ME operating primarily as a heteromer of α- and β-subunits. We found most angiosperm genomes maintain a 1:1 β*-NAD-ME*/α*-NAD-ME* (β/α) relative gene dosage, but with some notable exceptions mainly due to additional duplications of β-NAD-ME subunits. For example, a significantly high proportion of species with C_4_-NAD-ME-type photosynthesis have a non-1:1 ratio of β/α. In the Brassicales, we found C_4_ species with a 2:1 ratio due to a β*-NAD-ME* duplication (β*1* and β*2*); this was also observed in the C_3_ *Tarenaya hassleriana* and Brassica crops. In the independently evolved C_4_ species, *Gynandropsis gynandra* and *Cleome angustifolia*, all three genes were affected by C_4_ evolution with α- and β1-*NAD-ME* driven by adaptive selection. In particular, the β1-NAD-MEs possess many differentially substituted amino acids compared with other species and the β2-NAD-MEs of the same species. Five of these amino acids are identically substituted in β1-NAD-ME of *G. gynandra* and *C. angustifolia*, two of them were identified as positively selected. Using synteny analysis, we established that β*-NAD-ME* duplications were derived from ancient polyploidy events and that α*-NAD-ME* is in a unique syntenic context in both Cleomaceae and Brassicaceae. We discuss our hypotheses for the evolution of NAD-ME and its recruitment for C_4_ photosynthesis. We propose that gene duplications provided the basis for the recruitment of NAD-ME in C_4_ Cleomaceae and that all members of the *NAD-ME* gene family have been adapted to fit the C_4_-biochemistry. Also, one of the β*-NAD-ME* gene copies was independently co-opted for its function in the C_4_ pathway.

## Introduction

C_3_ photosynthesis (Bassham et al., 1954) relies exclusively on ribulose-1,5-bisphosphate carboxylase oxygenase (rubisco) for carboxylase activity and evolved early in the history of life (Hayes, 1994). Rubisco is a bifunctional enzyme that catalyzes both the carboxylation and the oxygenation of its substrate ribulose-1,5-bisphosphate. One of the products of the oxygenase activity, 2-phosphoglycolate, is a toxic metabolite (Anderson, 1971; Kelly and Latzko, 1976; González-Moro et al., 1997). As a selective response to rubisco’s promiscuity, plants evolved the energetically costly photorespiratory pathway (Maurino and Peterhansel, 2010). Until ∼400 million years ago (Mya), the rubisco oxygenase reaction was negligible due to elevated CO_2_ and low O_2_ levels in the atmosphere (Sage and Monson, 1999; Leakey and Lau, 2012). After this time, the onset of oxygenic photosynthesis introduced changes in atmospheric conditions such as high levels of O_2_ which led to significant levels of costly photorespiration. Some land plants evolved a carbon concentrating mechanism, known as the C4 photosynthetic pathway, which resulted in reduction of the high-levels of photorespiration (Hatch, 1971; Heckmann et al., 2013). Nearly all the independent C_3_ to C_4_ transitions are dated to the mid-Oligocene (25–30 Mya), a time that was proceeded by a massive depletion of atmospheric CO_2_ (Christin et al., 2008; Vicentini et al., 2008; Edwards et al., 2010). Under a wide range of environmental conditions, such as high temperatures, dryness, and high light intensities, plants possessing the C_4_ biochemical pump are more efficient in terms of water and nitrogen use (Furbank and Hatch, 1987).

The initial step of the C_4_ photosynthetic pathway is the fixation of inorganic carbon onto phosphoenolpyruvate (PEP) by PEP-carboxylase (PEPCase) to produce a four−carbon (C_4_) acid (Hatch, 1987; Kanai and Edwards, 1999). The C_4_ acid moves to the site of rubisco where specific decarboxylases release CO_2_ (Drincovich et al., 2011). The decarboxylation reaction also produces a three−carbon acid, which diffuses back to the site of PEPCase where it is recycled to PEP. The C_4_ cycle effectively acts as a CO_2_ pump, increasing CO_2_ levels around rubisco such that it nearly saturates the active site and thus reduces photorespiration to a minimal level (von Caemmerer, 2000).

Two major C_4_ photosynthetic metabolic routes, known as the NAD-malic enzyme (ME) and NADP-ME subtypes, are distinguished by the primary ME decarboxylase used (Maier et al., 2011). In grasses, either of these subtypes can also make use of the facultative activity of PEPCase (Wang et al., 2014). C_4_ photosynthesis is a complex convergent trait that arose independently in at least 66 plant linages (Sage et al., 2011, 2012), the majority of which use the NADP-ME subtype (Sage et al., 2011). The NAD-ME subtype is mostly found in eudicot species, where it is found in approximately 20 C_4_-linages (Sage et al., 2011).

In plants using the NADP-ME subtype of C_4_ metabolism, C_4_-NADP-ME is present as a unique plastidial isoform with tissue specific expression and special regulatory properties, such as having pH dependent changes in oligomerization and substrate inhibition (Detarsio et al., 2007; Alvarez et al., 2019). The underlying molecular determinants for the distinction of C_4_-NADP-ME from the non-photosynthetic isoform (nonC_4_-NADP-ME) were recently characterized (Alvarez et al., 2019). The nonC_4_-NADP-ME is present as multiple isoforms in higher plants, which are located to plastids and cytosol and show tissue specific expression (Saigo et al., 2004; Gerrard Wheeler et al., 2005; Detarsio et al., 2008; Gerrard Wheeler et al., 2008; Maurino et al., 2009).

In contrast to the well-described C_4_-NADP-ME, C_4_-NAD-ME has yet to be characterized at the molecular level. Early studies of *Amaranthus hypochondriacus* and various monocot species showed contradictory data for subunit composition and oligomeric states (Murata et al., 1989; Long et al., 1994). In plants, NAD-ME is exclusively present in mitochondria, where its core function is in L-malate respiration, as an associated enzyme of the tricarboxylic acid cycle (Grover et al., 1981; Artus and Edwards, 1985; Tronconi et al., 2008; Fuchs et al., 2020). In *Arabidopsis thaliana*, NAD-ME functions as a homo- and/or heterodimer of two distinct, homologs proteins (∼65% sequence identity): known as the α-subunits (to which AtNAD-ME1 belongs) and β-subunits (to which AtNAD-ME2 belong) and with molecular masses in the range of 58 and 63 kDa (Tronconi et al., 2008, 2010a). The α- and β-NAD-ME share only 40% of identity with the NADP-ME isoforms, owing to the fact that the *NAD-ME* and *NADP-ME* genes were acquired in independent evolutionary processes in plants (Tronconi et al., 2018).

Modifications in function and expression are key components of the evolutionary transition of several enzymes involved in the C_4_ carbon concentrating mechanism. Gene duplication was proposed as a precondition for the evolution of C_4_ activities, as it facilitates C_4_-specific adaptive changes in *cis*-regulatory control regions as well as in coding regions (Lynch and Conery, 2000; Moore and Purugganan, 2005). The evolution of C_4_-NADP-ME likely followed this process of gene duplication and neofunctionalization, starting from a plastidial non-C_4_ isoform-coding gene and including the acquisition of a bundle sheath cell-specific expression pattern (Maurino et al., 2001; Tausta et al., 2002; Saigo et al., 2004; Christin et al., 2009). The C_4_ specific isoforms of NADP-ME evolved at least five times independently in this way in grasses (Christin et al., 2009). In contrast, the evolutionary history of C_4_-NAD-ME remains elusive, as to date a gene coding for a C_4_-specific isoform has not been identified (Murata et al., 1989; Long et al., 1994).

Here, we aimed at elucidating the mechanism of recruitment of NAD-ME for its function in the C_4_ pathway by focusing on the eudicot family Cleomaceae. This family contains species spanning a developmental progression from C_3_ to C_4_ photosynthesis and at least three separate origins of C_4_ lineages (Feodorova et al., 2010; Koteyeva et al., 2011). Cleomaceae belongs to the order Brassicales and is a sister group of the Brassicaceae, which contains one of the best studied plant species, *Arabidopsis thaliana*, for which vast amounts of -omics data are available for comparative analyses. Cleomaceae and Brassicaceae share the At-beta whole-genome duplication (WGD) event, which was estimated to have occurred 75–100 Mya (Edger et al., 2015). The Cleomaceae and Brassicaceae lineages diverged 41 Mya and more recently underwent the independent At-alpha (23–34 Mya) and Cs-alpha (14–20 Mya) WGDs (Schranz and Mitchell-Olds, 2006; Barker et al., 2009). Despite different patterns of gene loss and retention and chromosomal rearrangements after polyploidy, the genomes of Cleomaceae and Brassicaceae species show detectable synteny (Schranz and Mitchell-Olds, 2006). Moreover, there exist no significant differences in gene copy numbers between C_3_ and C_4_ *Cleome* species (van den Bergh et al., 2014).

Our comprehensive analyses indicate that a duplication of the *NAD-ME* gene during the evolution of vascular plants resulted in two paralogs lineages, α*-* and β-*NAD-ME*, which were retained during seed plant evolution and diversification. We propose that the heteromeric assembly of NAD-ME was established by sub-functionalization of the duplicated NAD-ME genes. Later, neo-functionalization optimized the α- and β-NAD-ME functions and changes in the subunit-specific duplications provided the basis for the recruitment of NAD-ME in C_4_ biochemistry. We found that in Cleomaceae all *NAD-ME* genes were affected by C_4_ evolution, where one of the β*-NAD-ME* gene copies was co-opted for its function in the C_4_ pathway.

## Materials and Methods

### Sequence Retrieval

For species with entire genome information, NAD-ME coding sequences were extracted from primary gene models www.phytozome.net. Sequences from Cleomaceae species were acquired from transcriptome data (Kulahoglu et al., 2014; Mabry et al., 2019); in case of ***Gynandropsis gynandra*** (C_4_), ***Cleome angustifolia*** (C_4_), and ***Tarenaya hassleriana*** (C_3_) the sequences were verified and correctly assembled using cDNA-based sequencing as the transcriptomes showed misassembled transcripts for several NAD-ME genes. Sequences for Chara braunii, Azolla filiculoides, Salvinia cucullata, and Panicum miliaceum were identified from their respective genome publications (Li et al., 2018; Nishiyama et al., 2018; Zou et al., 2019). Additional fern sequences were extracted from available large-scale transcriptomic data (Shen et al., 2018). Ginkgo biloba, Amborella trichopoda, Taxus baccata, Pinus pinaster, Pinus sylvestris, and Pseudotsuga menziesii sequences were collected via PLAZA 3.0 (Proost et al., 2015). Accession numbers of all NAD-ME coding sequences used in this work are listed in Supplementary Table 1. BLASTP with the BLOSUM62 as default scoring matrix and a minimal e-value of 0.0001 was implemented to obtain homologs using AtNAD-ME1 (AT2G13560) and AtNAD-ME2 (AT4G00570) as query. All sequences were manually checked for correct translation start sites and the presence of conserved amino acid regions found in all NAD(P)-ME (Tronconi et al., 2018). Mitochondrial localization was verified using the program Target P (Emanuelsson et al., 2000).

### Multiple Sequence Alignments

A data set of the coding sequences was assembled using MEGA X (v.10.0.5) (Kumar et al., 2018). The sequences were then translated into amino acids and aligned using Muscle (Edgar, 2004) with the gap opening penalty value of −2.9 and without penalizing its extension. Once retranslated into nucleotides, the alignment was manually edited to select the most-reliable positions in the alignment, assisted by Gblocks^1^ and TrimAl^2^ programs. Since the different phylogenetic methods consider columns with gaps in different ways, we applied a stringent criterion by eliminating codons with coverage less than 95%. The final multiple sequence alignment (MSA) consisted of 240 coding sequences from 118 species with 1,731 nucleotides positions corresponding to 577 codons.

### Phylogenetic Analyses

Bayesian inference (BI) was performed using MrBayes 3.1.2 software (Ronquist and Huelsenbeck, 2003). Two parallel runs, each including four Metropolis-coupled Markov chain Monte Carlo (MC3) analyses, were run for 5,000,000 generations and sampled every 1,000 generations. This generated an output of 5,000 trees per run. A site-specific rate model (partition scheme) was used. The characters in the MSA were divided into three sets corresponding to the codon positions. Each position has its own rate labeled m1 in case of the first codons site, m2 in case of the second codons site and m3 in case of the third codons site. For the tree inferred from the third positions we defined a one-partition scheme by excluding the characters represented by the first and second sites in the MSA. For an efficient Metropolis coupling, an incremental heating scheme of three heated chains and one cold chain in each run was used with a temperature parameter setting of 0.1. The final average standard deviation of split frequencies was used as the convergence index (values <0.01 indicated good convergence). The convergence of clade posterior probabilities within and between runs was checked using the potential scale reduction factor. The initial 25% of the sampled trees for each MC3 run were discarded as “burn-in” and the post-burn-in trees from the two runs were integrated to generate a 50% majority-rule consensus tree. The percentage of samples recovering any particular clade in a BI analysis represents the posterior probability (BPP) of a clade. In all cases, a GTR (General Time Reversible) model with base frequencies gamma shape parameter (G) and proportion of invariants sites (I) was set. All the active parameters in the GTR + G + I model were optimized separately for each position of the codons. For analyses using third codon positions, the number of chains in each run of was increased from four to five due to convergence conflicts. Nodes with BPP values >90% were considered highly supported.

Maximum likelihood (ML) and neighbor joining (NJ) analyses on the whole data set were conducted using MEGA X (v.10.0.5). The goodness of fit of each model to the data was measured by the Bayesian information criterion (BIC) and the model with the lowest BIC score was considered the best description for a specific substitution pattern. The initial tree for the ML search was generated automatically by applying the NJ and BIONJ algorithms, and its branch lengths were adjusted to maximize the likelihood of the data set for that tree topology under the selected model of evolution. Heuristic searches were conducted with the initial tree based on the nearest neighbor interchange (NNI) search where the alternative trees differ in one branching pattern. Reliability of interior branches was assessed with 2,000 bootstrap (B) re-samplings. Nodes with MLB or NJB values 50–69% were regarded as weakly supported, 70–84% as moderately supported, and 85–100% as strongly supported (Hillis and Bull, 1993). The tree files were saved in Newick format (.nwk) containing all the relevant clade support values and branch length information. The trees were displayed using the FigTree v1.4.4 software and edited by rotating nodes and compressing lineages that were designated by their subdivision, class, order, or family names.

### Differential Substitution Analysis

A strictly differentially substituted position is one for which the NAD-ME sequences of the C_4_ species, *G. gynandra* and *Cleome angustifolia* (recently reclassified as *Coalisina angustifolia*), contained an identical amino acid, while a second, different amino acid was shared in all other NAD-ME sequences. For the differential substitution analysis, we used the whole data set of NAD-MEs of the Brassicales obtained in this work. MSAs of α- and β-NAD-MEs were computed using the MAFFT algorithm (v7.427) with the iterative refinement method L-INS-i (Katoh et al., 2005; Kuraku et al., 2013). The sequences were aligned using the online tool integrated MKT^3^. The best amino acid substitution model based on each MSA was estimated using MEGA X (v.10.0.5) (Kumar et al., 2018). We used the MSAs to identify amino acid positions that are strictly differentially conserved in the α-NAD-ME and β-NAD-ME sequences of the C_4_ species as previously performed by Alvarez et al. (2019).

### Synteny Analysis

To further investigate the evolutionary relationships and duplication of Brassicaceae and Cleomaceae β*-NAD-ME* and α*-NAD-ME* genes, syntenic analysis was performed with the SynFind tool using the default parameters (last algorithm, window size set to 40 genes and with a minimum number of collinear anchors of 4) (Tang et al., 2015). For this analysis, a limited set of representative taxa was used based on phylogenetic breadth (see Figure 3), presence of ancient polyploidy (i.e., *Brassica* and *Gynandropsis*) and the availability of high-quality annotated draft genomes. For Cleomaceae, the genomes of *T. hassleriana*, *G. gynandra*, and *Cleome violacea* were included. For Brassicaceae we included *Arabidopsis thaliana*, *Arabis alpina*, *Brassica rapa*, and *Eutrema salsugineum*. As an outgroup representative, we included *Citrus clementina*. For our SynFind analyses, we used either *G. gynandra*, *A. thaliana* or *C. violacea* orthologs of β-NAD-ME and α-NAD-ME genes. The results of our synteny analyses were compared and visualized using GEvo (Tang et al., 2015).

### Positive Selection Tests and Statistical Analysis

For testing sites that underwent positive selection during the evolution of C_4_ species in the α-NAD-ME and β-NAD-ME coding sequences, different site-class specific models were employed using the software codeml, implemented in the PAML package (Yang, 2007). Because no gene lineages leading to C_4_-specific NAD-MEs were identified, we conducted a site-class-specific approach. The models assume that the ω = dN/dS (non-synonymous to synonymous substitution rates) take a value of 1 under neutral evolution. Positive and purifying (negative) selection are indicated when ω > 1 and ω < 1, respectively. The codon substitution models were: M0 (one-ratio) M1a (nearly neutral), M2a (positive selection), M3 (discrete), M7 (beta), M8 (beta and ω > 1), and M8a (beta and ω = 1) (Yang et al., 2000). The fit of these models to the sequence data was compared using likelihood-ratio test (LRT). When an LRT test yielded a significant result for any of the pairwise comparisons, the Bayes empirical Bayes (BEB) method was used to identify amino acids residues that have evolved under selection. Posterior probability of 0.90 was selected as the standard threshold for identifying residues under selection (Scheffler and Seoighe, 2005).

Significance of deviations from 1:1 β-NAD-ME/α-NAD-ME was determined according to the Fisher’s exact test using the SigmaStat software (Systat Software, Inc.).

## Results

### Identification of Two Major Clades of NAD-ME Genes (α- and β) in all Seed Plants

To cover the early and late evolution of NAD-ME proteins of land plants (Embryophyta), we analyzed a dataset of 253 coding sequences (Supplementary Table 1) from genomes and assembled transcripts from liverworts (Hepaticophyta), mosses (Bryophyta), early branching vascular plants (Lycophyta), ferns (Filicopsida), gymnosperms, angiosperms, and streptophyte algae (Charophytes), with chlorophyte algae (Chlorophyta) as the out-group. The coding sequences were aligned and used for BI and ML analyses to infer the phylogenetic gene and protein trees.

**FIGURE 1:**
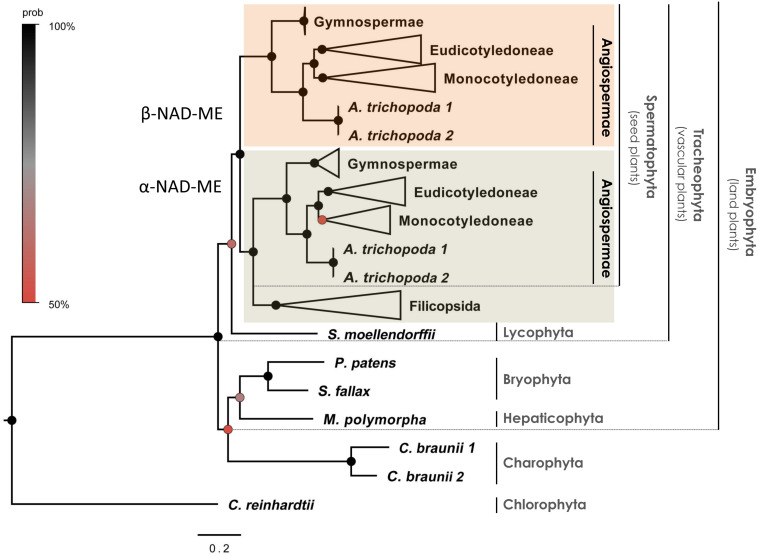
Phylogenetic gene tree of α- and β*-NAD-ME* in the green lineage. The phylogenetic relationships were inferred from coding sequences using BI. The main clades are compressed and designated by their subdivision, class, order or family names. Black circles highlight nodes with Bayesian Posterior Probability (BPP) values higher than 90%, gray circles indicate BPP values higher than 70% and red circles indicate BPP values higher than 50%. The three positions of codons were optimized separately (partitioned tree). Best-fit substitution model was a GRT + G submodel (BPP = 0.90 ± 0.01) with individual rates values: m1 = 0.42, m2 = 0.33, and m3 = 2.74 for the 1st, 2nd, and 3rd position of codons, respectively. The full tree is available in Supplementary Data 1.

We found all phylogenetic trees to be globally congruent regardless of the phylogenetic approach used (coding sequences-based BI tree in Figure 1 and Supplementary Data 1, coding sequences-based ML tree in Supplementary Data 2, and protein-based ML tree in Supplementary Data 3). The α- and β*-NAD-ME* genes formed well supported orthologs clades within the seed plants (spermatophytes). Both paralogs genes were retained across all gymnosperms and angiosperms analyzed and orthologs of α- and β-*NAD-ME* were not found in all other non-seed plants. In all the trees we found fern homologs confidently placed as a sister group to the α*-NAD-ME* of seed plants with Bayesian posterior probability (BPP) = 100% (Figure 1 and Supplementary Data 1) and MLB = 92 and 86% (Supplementary Data 2 and Supplementary Data 3, respectively). Similarly, we found no evidence for α- and β-*NAD-ME* duplicates in streptophyte algae, liverwort, mosses, nor the early branching tracheophyte *S. moellendorffii*. Given these data, the duplication of the *NAD-ME* gene and the fixation of both paralogs occurred at the foundation of the seed plants.

### Deviations From 1:1 β-*NAD-ME*/α-*NAD-ME* (β/α) Among NAD-ME of C_3_ and C_4_-Species

**FIGURE 2:**
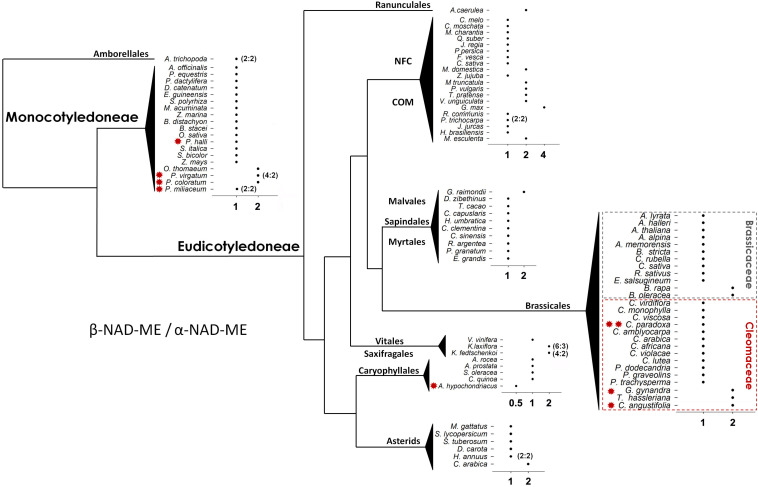
Scheme showing the ratio of β*-NAD-ME* to α-*NAD-ME* gene composition in Angiosperms. Most species have the same copy number (1 to 1). In the cases where the relative proportion and absolute composition differ, the absolute β to α gene dosage is indicated in parentheses. Schematic representation of the values with the species grouped according to their order and family interrelationships (APG IV, 2016). COM: Celastrales, Malpighiales, and Oxalideles. NFC (Nitrogen Fixing Clades): Rosales, Fabales, Cucurbitales, and Fagales. Red * and ** indicate C_4_-NAD-ME and C_3_–C_4_ photosynthetic metabolism, respectively. The scheme was created as a free art illustration where the lines do not represent branch lengths.

All 92 angiosperm species analyzed have retained orthologs of both NAD-ME α- and β coding genes and with most having a 1:1 β*-NAD-ME*/α-*NAD-ME* (β/α) relative gene dosage. However, changes in the relative genetic dosage could be identified (Figure 2). Twenty species have at least two β copies for each α gene, and only one species, *Amaranthus hypochondriacus* (Carophylles), has two α genes for one β (Figure 2). The most pronounced change was found in *Glycine max* with a β/α gene ratio of 4:1. In eudicots, the NFC (Nitrogen Fixing Clade including: Rosales, Fabales, Cucurbitales, and Fagales) and COM (Celastrales, Oxalideles, and Malpighiales) clades contain 42% of the species with a non-1:1 ratio. Species having C_4_-NAD-ME photosynthesis, with the exception of *Panicum halli* and *Panicum miliaceum* (Poaceae), have a β/α relation deviating from 1:1. This proportion (71.5%) is significantly higher than that observed for non-C_4_ NAD-ME species (18.6%) (Fisher’s exact test, *P* = 0.006). The C_3_–C_4_ intermediate *Cleome paradoxa* does maintain the 1:1 β/α ratio.

### Evolution of α- and β-NAD-ME Proteins in Cleomaceae and Brassicaceae

Gene duplication plays a critical role in generating the diversity needed for the evolution of protein through neo-functionalization. We wanted to investigate the role of gene duplication to the evolution of NAD-ME type C_4_ photosynthetic metabolism in Eudicot species. NAD-ME has been independently co-opted for C_4_ photosynthesis in Caryophyllales and Brassicales species. Thus, we further investigated the pattern of the NAD-ME protein evolution in the Cleomaceae (having both C_3_ and C_4_ species) and in its sister-family the Brassicaceae (including Arabidopsis and Brassica crops). Most species in both families have a 1:1 ratio of β/α relative gene dosage (Figure 2). However, both families possess species carrying an additional copy of the β*-NAD-ME* gene (β*1* and β*2*) (Figure 2) that correlate with mesopolyploidy events. In Cleomaceae, the β*-NAD-ME* gene duplication is found in the C_3_ species *T. hassleriana* and the C_4_ species *C. angustifolia* and *G. gynandra* (Figure 2). *T. hassleriana* and *G. gynandra* share the Th-alpha ancient polyploidy event, but *C. angustifolia* underwent an independent polyploidy event (Mabry et al., 2019). In Brassicaceae, both *B. rapa* and *B. oleracea* have duplicated β*-NAD-ME* gene associated with the Br-α genome triplication.

**FIGURE 3:**
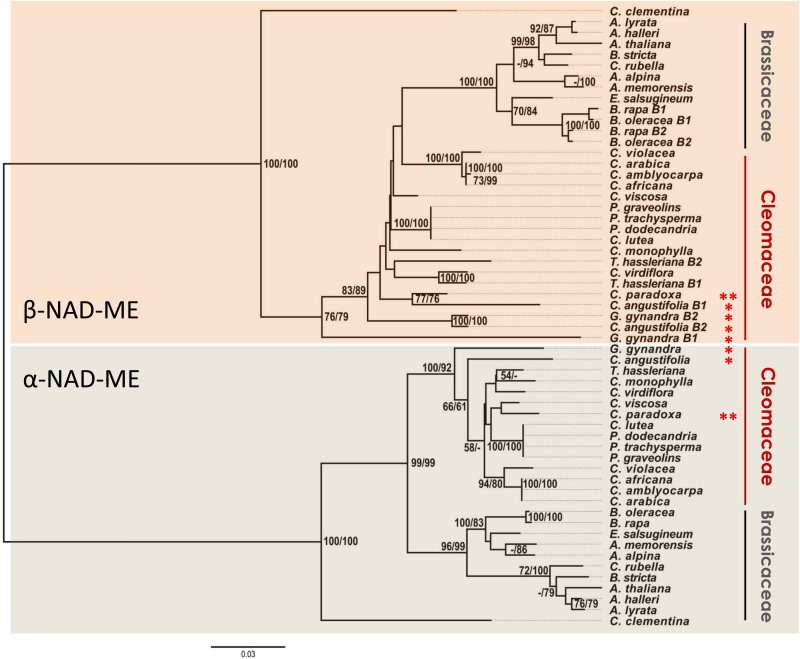
Phylogenetic gene tree of NAD-ME proteins of Cleomaceae and Brassicaceae. The evolutionary history was inferred using the ML and NJ methods. The NJ optimal tree with the sum of branch length = 1.58 is shown. The tree is drawn to scale, with branch lengths measured in the number of substitutions per site. The evolutionary distances were computed using the JTT matrix-based method with a discrete Gamma distribution to model evolutionary rate differences among sites (G, parameter = 0.52). Support values of 2,000 bootstrap replicates are given as MLB/NJB next to the branches when greater than 50%. The analysis involved 57 amino acid sequences and there were 227 parsimony-informative sites over a total of 597 positions (less than 40%) in the final dataset. Red * and ** indicate C_4_ and C_3_–C_4_ photosynthetic metabolism, respectively. *Citrus clementina* NAD-ME isoforms were used as out groups. The full trees are available in Supplementary Data 4 and Supplementary Data 5.

The NJ (Figure 3 and Supplementary Data 4) and ML (Supplementary Data 5) phylogenetic protein tree topologies were very consistent despite the low support values of some branches (MLB and NJB <50%). For closely related species, the protein-based tree has fewer informative sites than the CDS-based tree (Figure 1). However, we were interested in evaluating unusual branch positions and lengths that would suggest evolutionary rate shifts and altered amino acidic sequences. In the Brassicaceae, the relationships of the orthologs in the α-NAD-ME and β-NAD-ME clades agree with recent assessments of the species phylogenetic relationships (Nikolov and Tsiantis, 2017; Bayat et al., 2018). Conversely, α- and β-NAD-ME protein trees of Cleomaceae are incongruent with current species trees (Feodorova et al., 2010; Patchell et al., 2014).

Regarding the α-NAD-ME orthologs, we recovered the core *Cleome sensu stricto* (*s. str.*) (*C. violacea*, *C. arabica*, *C. africana*, and *C. amblyocarpa*) and the clade containing *Polanisia* species with well-supported MLB and NJB values (Patchell et al., 2014). However, the α-NAD-ME of the C_4_ species *G. gynandra* and *C. angustifolia* were placed as the base of the clade within the Cleomaceae (Figure 3), which disagrees with known species relationships.

In the β-NAD-ME clade, the conflicting pattern of protein relationships from known species relationships is even more pronounced. Here, the β-NAD-ME of Cleomaceae was recovered as a non-monophyletic group with Brassicaceae as a sister group of the Cleome *s. str.* (Figure 3). Within the Brassicaceae we recovered the same groups as described in the α-NAD-ME clade. Even more so, the β-NAD-ME of the C_4_ species *G. gynandra* and *C. angustifolia* appear as first-branching lineages that precede the formation of Brassicaceae. This whole pattern could be explained by an unrealistic evolutionary scenario in which the β-NAD-ME of the C_4_ species *G. gynandra* and *C. angustifolia* are encoded by ancient genes retained only in the genome of these species. Instead, the differences in rates of molecular evolution within the Cleomaceae are most probably due to selective pressures on the NAD-ME genes of the C_4_ species. In addition to the incongruent positioning in the protein-based trees, the α- and β-NAD-ME of the C_4_ species show branch lengths that do not correlate with those of the C_3_ orthologs in Cleomaceae (Supplementary Data 4). At least in *G. gynandra*, such a high number of non-synonymous substitutions cannot be a consequence of pseudogenization, as α- and β*2-NAD-ME* transcripts are highly abundant in leaves (Brown et al., 2011; Kulahoglu et al., 2014).

### Congruency Between NAD-ME Gene-Based Trees and Cleomaceae Species Trees

We hypothesize that evolutionary forces driving the evolution of C_4_ in the Cleomaceae could be responsible for the incongruent NAD-ME protein-based phylogenetic trees. To address this hypothesis, we inferred phylogenetic trees from the third position of the codons, which is considered as a nearly neutral marker (Christin et al., 2007; Hilu et al., 2014). We conducted this analysis focused on the NAD-ME coding sequences of the Rosids, a well-defined monophyletic group capable of reconstructing true phylogenetic relationships given the high phylogenetic signals and low homoplasy (Hilu et al., 2014).

**FIGURE 4:**
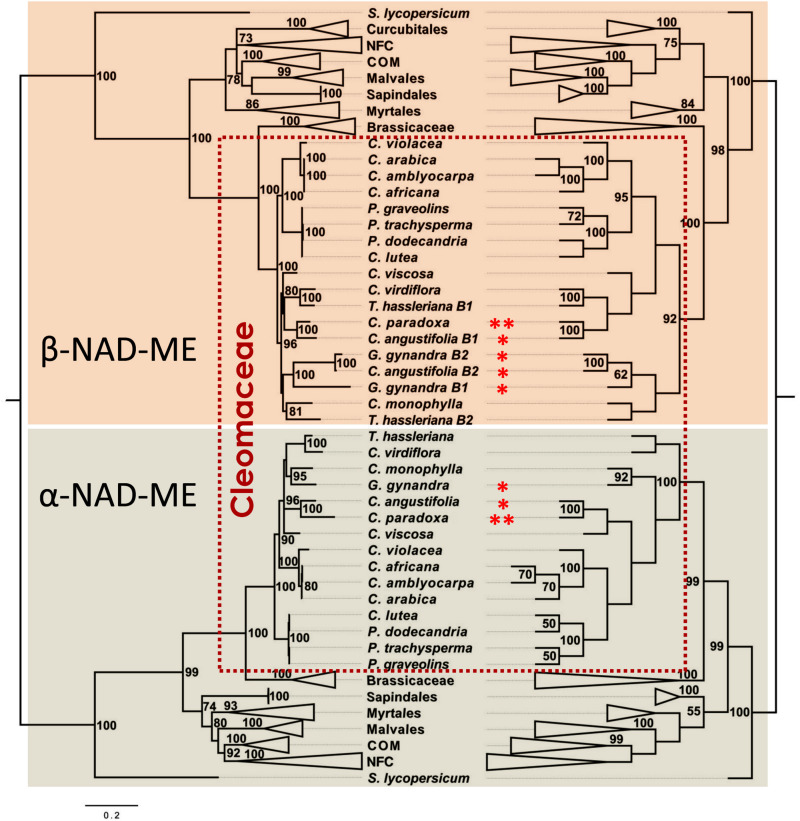
Evolutionary history of *NAD-ME* genes in Cleomaceae deduced from third position of the codons. Bayesian **(left)** and Maximum Likelihood **(right)** phylogenetic tree of NAD-ME of the Rosid lineage. Most of the clades are compressed and designated by their Order or Family names. BPP and MLB values higher than 70 and 50%, respectively, are given next to the branches. In the BI analysis, the tree is drawn to scale with branch lengths measured in the number of substitutions per site. In the ML analysis, the bootstrap consensus tree (dendogram) inferred from 2,000 replicates is taken to represent the evolutionary history of the taxa, in which the partitions reproduced in less than 50% of the bootstrap replicates are collapsed. In both analyses, the best-fit substitution model was a GRT + G (3.70) model involving 124 nucleotide sequences and a total of 497 positions in the final dataset. * and ** indicate C_4_ and C_3_–C_4_ photosynthetic metabolism, respectively. *Solanum lycopersicum* NAD-MEs coding sequences were used as out groups. The full trees are available in Supplementary Data 6 and Supplementary Data 7.

The BI (Figure 4, right and Supplementary Data 6) and ML (Figure 4, left and Supplementary Data 7) phylogenetic trees deduced from unconstrained sites were widely congruent with well-supported nodes. In the α*-NAD-ME* clade, we recovered the expected topology based on published species phylogenies inferred from plastid, mitochondrial and nuclear markers (Feodorova et al., 2010; Patchell et al., 2014). We found that *Polanisia* and the *Cleome s. str.* species cluster as basal lineages of C_3_ species. The C_4_ species *G. gynandra* and *C. angustifolia* form paraphyletic groups, with the C_3_-C_4_ intermediate *C. paradoxa* confidently placed in the Angustifolia clade and *G. gynandra* nested close to the African species *C. monophylla* (Figure 4). Finally, the C_3_ species *T. hassleriana* and *C. virdiflora* confidently branch together as members of the *Tarenaya* clade. For the β*-NAD-ME* gene tree, the topology of the *Polanisia* and *Cleome s. str.* clades is consistent with species relationships. Again, *Polanisia* and the *Cleome s. str.* cluster together as first-branching C_3_ species (Figure 4). However, β*-NAD-ME* duplicated genes of *T. hassleriana*, *G. gynandra*, and *C. angustifolia* do not group as paralogs gene pairs or a cluster of orthologs. Instead a complex species-dependent arrangement of β*1* and β*2* genes is observed. In the C_3_ *T. hassleriana*, the β*1* copy nests with its *C. virdiflora* ortholog, in line with the α*-NAD-ME* grouping, and *Th*β*2-NAD-ME* is found in a separate clade with *C. monophylla*. A plausible explanation is that the duplication of β-*NAD-ME* occurred in a common ancestor to these three C_3_ related species and, after speciation, *C. virdiflora* lost the β*1* gene and *C. monophylla* lost the β*2* gene. In the C_4_ species, the β*1* gene of *C. angustifolia* groups with the *C. paraxoda* ortholog, in concordance with the α*-NAD-ME* lineage, but the β*2* copy groups with β*2* of *G. gynandra*. Finally, β*1* of *G. gynandra* is placed basal to the *Gg*β*2-NAD-ME–Ca*β*2-NAD-ME* cluster (Figure 4). Hence, the β*-NAD-ME* duplication and subsequent loss of one copy seems to be a possible common phenomenon in Cleomaceae, which overshadows the true gene relationship. However, because of the essential role of NAD-ME in C4 photosynthesis, we are confident to propose that strong selective pressures accelerated the mutational rate of the α- and β*-NAD-ME* genes, as the species tree was globally recovered when nearly neutral markers are used.

### Synteny Analysis of β-NAD-ME and α-NAD-ME Genes

**FIGURE 5:**
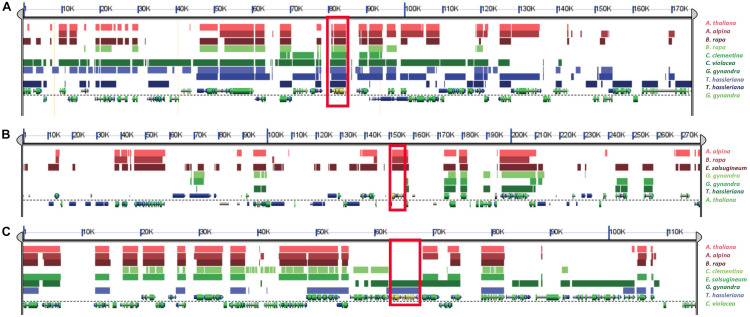
Syntenic relationships of β*-NAD-ME* and α*-NAD-ME* genes across Cleomaceae, Brassicaceae and outgroup genomes using SynFind and GEvo analysis. **(A)** A search for syntenic regions based on the β*-NAD-ME1* gene in *G. gynandra* (as reference region) identified syntenic homologs in all species (shown in red box), including the outgroup species *C. clementina*. Also, all duplicated copies found by phylogenetic analysis of β*-NAD-ME*, for example in *B. rapa* and *T. hassleriana*, were also syntenic which supports that they are derived from ancient polyploidy events. **(B)** A search for syntenic regions based on the *A. thaliana* (Brassicaceae) copy of α-NAD-ME identified only single copy syntenic homologs (shown within red box) in other Brassicaceae species (*A. alpina*, *E. salsugineum*, and *B. rapa*) but not with Cleomaceae species (*C. violacea*, *G. gynandra*, and *T. hassleriana*) nor the outgroup species *C. clementenia*. Similarly, **(C)** A search for syntenic homologs of the *C. violacea* α*-NAD-ME* gene (shown within red box) found them only in the other Cleomaceae species (*G. gynandra* and *T. hassleriana*) but not with Brassicaceae or the outgroup species. These combined results **(B, C)** suggest independent translocations of α*-NAD-ME* genes in the Brassicaceae and Cleomaceae to new genomic contexts.

The analysis of syntenic relationships of β*-NAD-ME* and α*-NAD-ME* genes in the genomes of Cleomaceae and Brassicaceae allowed us to clarify the evolutionary dynamics and duplication histories in these two families. We found that all retained copies of β*-NAD-ME* genes are syntenic across both families and with outgroup species (Figure 5A). Furthermore, it was evident that duplicate copies of β*-NAD-ME* in *Tarenaya, Gynandropsis*, and *Brassica* can be attributed to the known ancient polyploid histories of these species as duplicate copies of the genes were found in larger intra-genomic blocks. We also detected syntenic regions in all species derived from ancient polyploidy events, such as the At-alpha event at the origin the Brassicaceae, where a duplicated copy of β*-NAD-ME* was lost due to genome fractionation/gene loss processes that are known to have occurred.

We found a very different evolutionary pattern when examining the syntenic relationships for α*-NAD-ME* homologs. We found that α*-NAD-ME* genes are in a different genomic context in both families compared to all other angiosperms and in a specific and conserved context in Brassicaceae (Figure 5B) and in Cleomaceae (Figure 5C). The α*-NAD-ME* homologs are all syntenic with one another within the Brassicaceae, but not with Cleomaceae nor out-group species. Thus, we conclude that the ancestral α*-NAD-ME* gene transposed to a new genomic context in Brassicaceae after its divergence from the ancestor of Cleomaceae, potentially associated with the dramatic genome repatterning that would have occurred after the At-alpha WGD event. Whereas all three examined Cleomaceae species (*C. violacea*, *G. gynandra*, and *T. hassleriana*) α*-NAD-ME* orthologs are syntenic. *C. violacea* lacks evidence of the ancient polyploidy event shared between *G. gynandra* and *T. hassleriana*. Thus, the ancestor of all Cleomaceae α*-NAD-ME* must have transposed before the splitting of these two lineages (perhaps even earlier during the evolution of the Brassicales). The unique transpositions of α*-NAD-ME* to independent new genomic contexts may have had an impact of the expression and function of the gene in both Brassicaceae and Cleomaceae species compared to other eudicot species. As mentioned above, all Brassicaceae and Cleomaceae species retain only a single copy of α*-NAD-ME* and consistently we do not detect any retained and syntenic gene duplicates due to ancient genome duplications. Like with β*-NAD-ME*, we detected syntenic regions derived from ancient polyploidy events where a duplicated copy of α*-NAD-ME* was lost due to genome fractionation.

### Amino Acid Substitutions in NAD-ME During the Evolution of C_4_ Photosynthesis in Cleomaceae

We found a high number of non-synonymous substitutions in the β-NAD-ME sequences of the C_4_ species (Figure 3). We further analyzed the patterns of amino acid positions in the β-NAD-ME sequences that accumulated changes during C_4_ evolution in Cleomaceae compared to all Brassicales β-NAD-MEs in a MSA (Supplementary Figure 1). We found that 393 of 579 amino acids (∼68%) are identical in all mature β-NAD-ME protein sequences (Supplementary Figure 1). The most diverged protein sequences are the sequences of C_4_ species *G. gynandra* β1-NAD-ME (186 amino acids, ∼32%) and *C. angustifolia* β1-NAD-ME (158 amino acids, ∼27%). Interestingly, β1-NAD-MEs of *G. gynandra* and *C. angustifolia* share many changed amino acids and both sequences differ only in 83 positions.

To identify amino acid residues potentially involved in C_4_-optimization, we compared the NAD-MEs of the Brassicales in the MSA searching for differentially substituted amino acids in the NAD-ME protein sequences of the C_4_ species. A differentially substituted amino acid is a position in the MSA at which a NAD-ME protein of a C_4_ species has an amino acid that differs from all other NAD-ME proteins, which all share the same amino acid (Alvarez et al., 2019).

We found three amino acid positions, F127, Q205, and N466 (numbered according to the full-length sequence of *G. gynandra*; Supplementary Figure 2), strictly differentially substituted in both *G. gynandra* and *C. angustifolia* α-NAD-ME proteins (Supplementary Figure 2, orange amino acids). Interestingly, we found that 36 amino acids are differentially substituted in the β1-NAD-ME from *G. gynandra* (Supplementary Figure 1, light blue + orange amino acids). Similarly, we identified 13 amino acids changes in the β1-NAD-ME of *C. angustifolia* (Supplementary Figure 1, dark blue + orange amino acids). Interestingly, 5 amino acid positions, V131, S132, H195, V297, and K605 (numbered according to the full-length sequence of *G. gynandra*) are identically substituted in both β1-NAD-ME of the C_4_ species *G. gynandra* and *C. angustifolia* (Supplementary Figure 1, orange amino acids). This kind of amino acid replacement was not observed in the β2-NAD-ME isoforms of the C_4_ species.

The strictly differentially substituted amino acids in the α-NAD-ME and β1-NAD-ME of *G. gynandra* and *C. angustifolia* suggest that these amino acids probably evolved under positive selection. To address this, we carried out model tests to identified residues that underwent adaptive changes in the α-NAD-ME and β1-NAD-MEs of the C_4_ species. The models were optimized using the tree topology inferred from third positions of codons (Figure 4, right) and the mature α-NAD-ME and β-NAD-ME coding sequences of Cleomaceae in the MSA. For both paralogs, one model (M8), allowing a proportion of codons evolving under positive selection, provided better fit than the null model (M7) (Supplementary Tables 2,3). We found five amino acid positions having a posterior probability greater than 0.9 in the α-NAD-MEs: 80, 205, 423, 500, and 587 (numbered according to the full-length sequence of *G. gynandra*; Supplementary Figure 2). The position 205 corresponds to the differentially substituted Q205 in the α-NAD-ME of *G. gynandra* and *C. angustifolia* (Supplementary Figure 2). For the β1-NAD-MEs, three amino acid positions had a posterior probability greater than 0.9: 132, 297, and 395 (numbered according to the full-length sequence of *G. gynandra*, Supplementary Figure 1). The positively selected positions 132 and 297 correspond to the differentially substituted S132 and V297 residues in β1-NAD-ME of *G. gynandra* and *C. angustifolia*.

## Discussion

### Sub-Functionalization Set Up the Heteromeric Assembly of NAD-ME and Neo-Functionalization Optimized the α- and β-Subunit Functions

We identified a duplication of *NAD-ME* in vascular plants that generated the two paralogs lineages: α- and β*-NAD-ME*. All seed plants examined maintained at least one α*-NAD-ME* and one β*-NADME* –homolog (Figure 1 and Supplementary Data 1), with most species maintaining a 1:1 α/β relative gene dosage (Figure 2). This is a strong indication that the α- and β-NAD-ME homologs diversified and evolved non-redundant, alternative and critical (housekeeping) functions. In higher plants the heteromeric assembly of NAD-ME is most probably a requirement to fully fit into the central carbon metabolism, and specific duplication of the NAD-ME subunit-coding genes are evolutionarily allowed.

**FIGURE 6:**
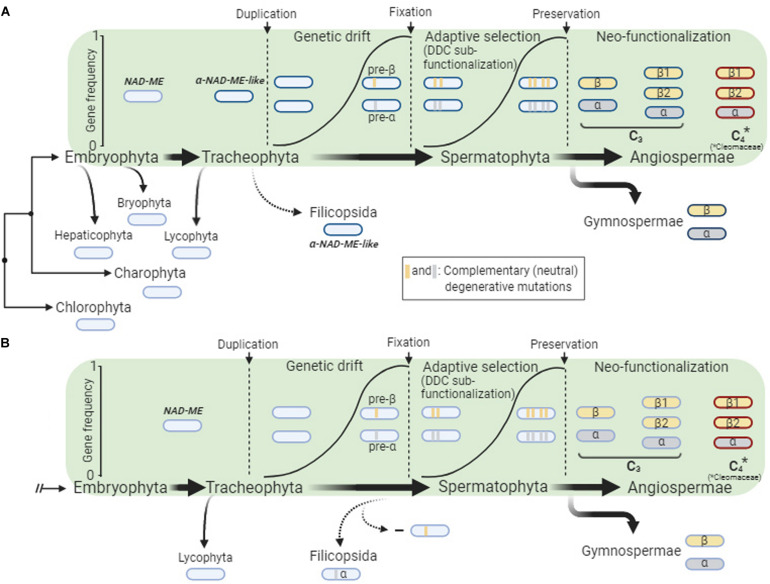
Scheme showing our hypothesis for the evolution of the α*-NAD-ME* and β*-NAD-ME* genes in seed plants. The green panel shows the general course of the gene duplication event and the frequency of the both genes in the population until their fixation (genetic drift) and preservation (adaptive selection). The *NAD-ME* duplication took place late during the evolution of vascular plants, as the α- and β*-NAD-ME* paralogs were not found in Lycophytha, an early branching division of Tracheophyta. After that, one possible scenario is that one α*-NAD-ME-like* gene lineage was duplicated and fixed in seed plants but not in ferns, which kept a single copy **(A)**. Alternatively, the NAD-ME duplication could have occurred before Filicopsida and Spermatophyta split and the pre-β*-NAD-ME* copy was lost in ferns, as no selective pressure was exerted at that point **(B)**. In seed plants, adaptive selection through a duplication-degeneration-complementation (DDC) sub-functionalization process gave rise to the α- and β-*NAD-ME* gene lineages. Once preserved, both paralogs were double down and split, providing raw material for functional innovations.

The α*-NAD-ME* and β*-NAD-ME* gene lineages evolved by duplication of an ancestral *NAD-ME* gene that occurred late during the evolution of vascular plants, as the paralogs were not found in the early branching tracheophyte *S. moellendorffii* (Figure 1). The well supported positioning of the single *NAD-ME* genes of ferns as a sister group of spermatophytes α-NAD-ME (Figure 1, Supplementary Data 2 and Supplementary Data 3) suggests two alternative scenarios for the duplication of *NAD-ME* (Figure 6): (A) an ancestral α*-NAD-ME-like* gene likely diverged in Tracheophytes after the Lycophytes separation. This ancestral gene duplicated at the origin of the seed plants giving rise to *pre*-α*-NAD-ME* and *pre-*β*-NAD-ME* paralogs sequences, which afterward diverged from the ancestral α*-NAD-ME-like* gene. Finally, the paralogs were fixed (possibly by genetic drifts) and adaptive selection preserved them in seed plants. In this scenario, a primitive α*-NAD-ME-like* gene was retained in lycophytes; alternatively, (B) a *NAD-ME* duplication giving rise to *pre*-α*-NAD-ME* and *pre-*β*-NAD-ME* paralogs took place in Tracheophytes directly after the Lycophytes separation. The pre-β*-NAD-ME* was then lost in the ferns before adaptive selection could preserve both genes (Figure 6). Instead, *pre*-α*-NAD-ME* and *pre-*β*-NAD-ME* diverged from each other and were preserved as the α- and β*-NAD-ME* paralogs in seed plants.

Four major models have been proposed for preservation of duplicated genes: neo-functionalization, specialization, dosage selection and duplication-degeneration-complementation (DDC) sub-functionalization (Conant and Wolfe, 2008; Innan and Kondrashov, 2010). Neo-functionalization and specialization provide an explanation for the cases where after fixation by genetic drift, one or both duplicate genes diverge from the ancestral function (e.g., in expression patterns, substrate specificities, etc.). The dosage selection model supposes that the new duplicated gene evolves by positive selection (e.g., the increase in the amount of protein is beneficial; the new copy emerges with a novel function or by shielding against deleterious mutations). The DDC sub-functionalization is a conservative evolutionary model that assumes that the functions of an ancestral gene have been neutrally divided among the daughter copies due to complementary degenerative mutations: neither copy is able to fulfill the original functions on their own.

Previous findings support the DDC sub-functionalization process by which the duplicated α- and β*-NAD-ME* copies could be preserved: (i) To fulfill the housekeeping function in L-malate respiration in seed plants, the NAD-ME is predominantly assembled as an heteromer of α- and β-subunits, with both catalyzing the same reaction (Tronconi et al., 2008, 2010b); (ii) both α*-NAD-ME* and β*-NAD-ME* genes are constitutively expressed and coordinately regulated and the encoded proteins accumulate at similar levels in the most plant tissues (Tronconi et al., 2008, 2010b; Fuchs et al., 2020); (iii) knockout lines of Arabidopsis lacking either α-NAD-ME or β-NAD-ME show residual NAD-ME activities, which in sum do not reach the activity measured in wildtype. Moreover, the mutants plants lacking β-NAD-ME retain less than 10% of the total NAD-ME activity (Tronconi et al., 2008; Brown et al., 2011); (iv) The identification of coevolved connected amino acidic residues belonging to the α-NAD-ME and β-NAD-ME subunits (Tronconi et al., 2018) indicates that compensatory neutral mutations marked the evolution toward a functional heteromeric NAD-ME in higher plants.

Following the preservation phase, neo-functionalization can occur in one or both duplicated genes (Conant and Wolfe, 2008). In this regard, the comprehensive characterization of *A. thaliana* NAD-ME indicated that the α-NAD-ME and β-NAD-ME homodimers and the α/β-NAD-ME heterodimer behave differently in terms of catalytic mechanism, interaction with the substrates and allosteric regulation (Tronconi et al., 2008, 2010a, b, 2015), pointing out an adaptive advantage in terms of metabolic flexibility. This metabolic flexibility might have played a role in the adaption of a C_4_-specific version of the NAD-ME. Moreover, α- and β-NAD-ME differentially accumulate in the separate components of the floral organ (Tronconi et al., 2010b). In sepals, the α-NAD-ME is present at a slightly higher proportion than β-NAD-ME. On the other hand, β-NAD-ME is the only protein present in anthers. All these observations suggest that NAD-ME activity may be regulated by variations of the native association *in vivo*, rendering enzymatic entities with distinct allosteric regulation to fulfill additional metabolic roles (Maurino et al., 2009; Tronconi et al., 2010b; Maier et al., 2011).

### Changes in the Subunit-Specific Duplications Provide the Basis for the Recruitment of NAD-ME in C_4_ Biochemistry

In multiple families of the angiosperms we found a higher number of *NAD-ME* genes coding for β-NAD-ME (20 species) than for α-NAD-ME (one species) (Figure 2). This indicates that if small-scale gene duplications or WGDs occurred, the gene coding for a β-NAD-ME is tolerant of independent copy number variation (possibly fixed by genetic drift) while the α-*NAD-ME* gene is under evolutionary pressure to return to a 1:1 β/α relative status. It seems that the α*-NAD-ME* duplication imparts a detrimental effect or does not increase plant fitness and thus, the duplicated gene is not fixed in the population. A new copy can escape the negative effect of the dosage if during the fixation phase (Figure 6) a mutation conferring an adaptive advantage arises (Conant and Wolfe, 2008). This can explain the observation that the C_4_-NAD-ME species *A. hypochondriacus* has retained two α-*NAD-ME* genes (Figure 2). Probably, shortly after the duplication, a copy quickly diverged from the original function by neo-functionalization to fit to the C_4_ biochemistry.

The significantly higher proportion of C_4_-NAD-ME species possessing an additional *NAD-ME* gene copy (Figure 2) is consistent with the general notion that gene duplication is a precondition for the evolution of the C_4_ functions (Lynch and Conery, 2000; Moore and Purugganan, 2005). For other C_4_ genes, one copy of a duplicated gene is neo-functionalized without affecting the other members of the genetic family (Christin et al., 2013). However, in the C_4_ species of Cleomaceae all three genes were potentially affected by adaptive selection as suggested by the inconsistencies observed in the protein-based tree topology (Figure 3). This could be rectified for the α*-NAD-ME* genes, albeit partially for the β*-NAD-ME* genes, when a nearly neutral marker (third codon positions) was used (Figure 4). Higher amino acid substitution rates are associated with an accelerated evolution or potential positive selection due to neo-functionalization of the resulting protein sequence. Neo-functionalization from a duplicated gene is a classic driver of protein evolution.

We detected a high proportion of base substitutions in the Cleomaceae C_4_ species β*-NAD-ME* genes. The β*-NAD-ME* genes are duplicated in the Cleomaceae C_4_ species *C. angustifolia* and *G. gynandra* and the C_3_ species *T. hassleriana*, and in the Brassicaceae *B. rapa* and *B. oleracea*. Synteny analysis clearly showed that duplication of β*-NAD-ME* in both families (Figure 5A) was due to the ancient polyploidy events, such as the *Brassica* lineage genome triplication and the *Tarenaya* WGD that is shared with *G. gynandra* (Cheng et al., 2014; van den Bergh et al., 2014). Importantly, changes in copy number of genes can maintain dosage-balance relationships if generated by polyploidy events. Nevertheless, only the β1-NAD-ME of both C_4_ species has accumulated a high number of amino acid changes. Intriguingly, five positions show the same amino acid changes in both β1-NAD-ME of these C_4_ (Supplementary Figure 1), two of which (S132 and V295) evolved under positive selection (Supplementary Table 3). We hypothesize that these amino acids play a role in the C_4_ function of NAD-ME, as *C. angustifolia* and *G. gynandra* belong to different C_4_ lineages and the five amino acids are differentially conserved in the β-NAD-ME of C_3_ Cleome species, Brassicales β-NAD-ME or the respective β2-NAD-ME of *C. angustifolia* and *G. gynandra*. Interesting, four of these five amino acid are involved in substrate coordination or are located in the L-malate binding domain (Chang and Tong, 2003). Finally, we found three differentially conserved amino acid substitutions in the α-NAD-ME of the C_4_ species (Supplementary Figure 2), one of them (Q205) identified as an adaptive change (Supplementary Table 2). This adaptive and differentially conserved position in the α-NAD-ME is neither part of the enzymatic active center nor was shown to participate in the enzymatic mechanism of reaction (Chang and Tong, 2003). Because of the heteromeric NAD-ME assembly, this substitution most likely represents a change necessary to compensate for the evolutionary changes in the β1-NAD-ME, or enable another kind of function that is necessary in the adaption of NAD-ME in the C_4_ context.

## Conclusion

It appears that the genes encoding C_4_ enzymes evolved by simply duplication of an original metabolic enzyme and further neo-functionalization (Christin et al., 2013; Ludwig, 2016; Alvarez et al., 2019). NAD-ME turns out to be an exceptional case, probably due to its heteromeric structure. NAD-ME followed an intricated molecular mechanism of evolution marked by sub-functionalization and differences in the frequency of α- and β*-NAD-ME* gene duplication (Figure 6). Future work should focus on how NAD-ME in C4 plant mitochondria has been adapted to perform both housekeeping and C_4_-associated functions.

## Data Availability Statement

All datasets presented in this study are included in the article/Supplementary Material.

## Author Contributions

VM conceived the project in active discussion with all co-authors. MT and MH performed the phylogenetic analysis. MS performed the syntenic analysis. All authors contributed to data analysis and writing the manuscript. All authors contributed to the article and approved the submitted version.

## Conflict of Interest

The authors declare that the research was conducted in the absence of any commercial or financial relationships that could be construed as a potential conflict of interest.

## Footnotes

1 http://molevol.cmima.csic.es/castresana/Gblocks.html

2 http://trimal.cgenomics.org/

3 https://imkt.uab.cat
