# Supplementary figures and images for: Independent Recruitment of Duplicated β-Subunit-Coding NAD-ME Genes Aided the Evolution of C4 Photosynthesis in Cleomaceae
Source: Front Plant Sci. 2020 Oct 6;11:572080. doi: 10.3389/fpls.2020.572080 (PMC7573226; doi:10.3389/fpls.2020.572080)

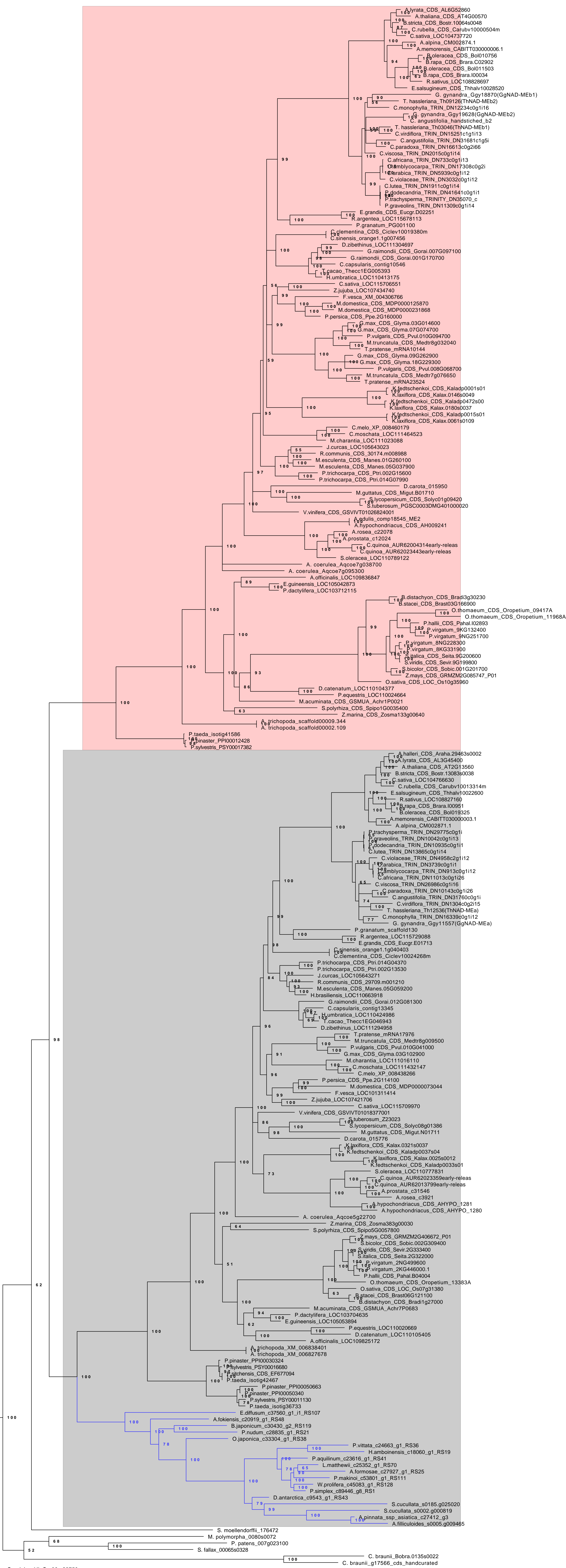

Supplement: Supplementary file 1 [file Image_1.pdf]

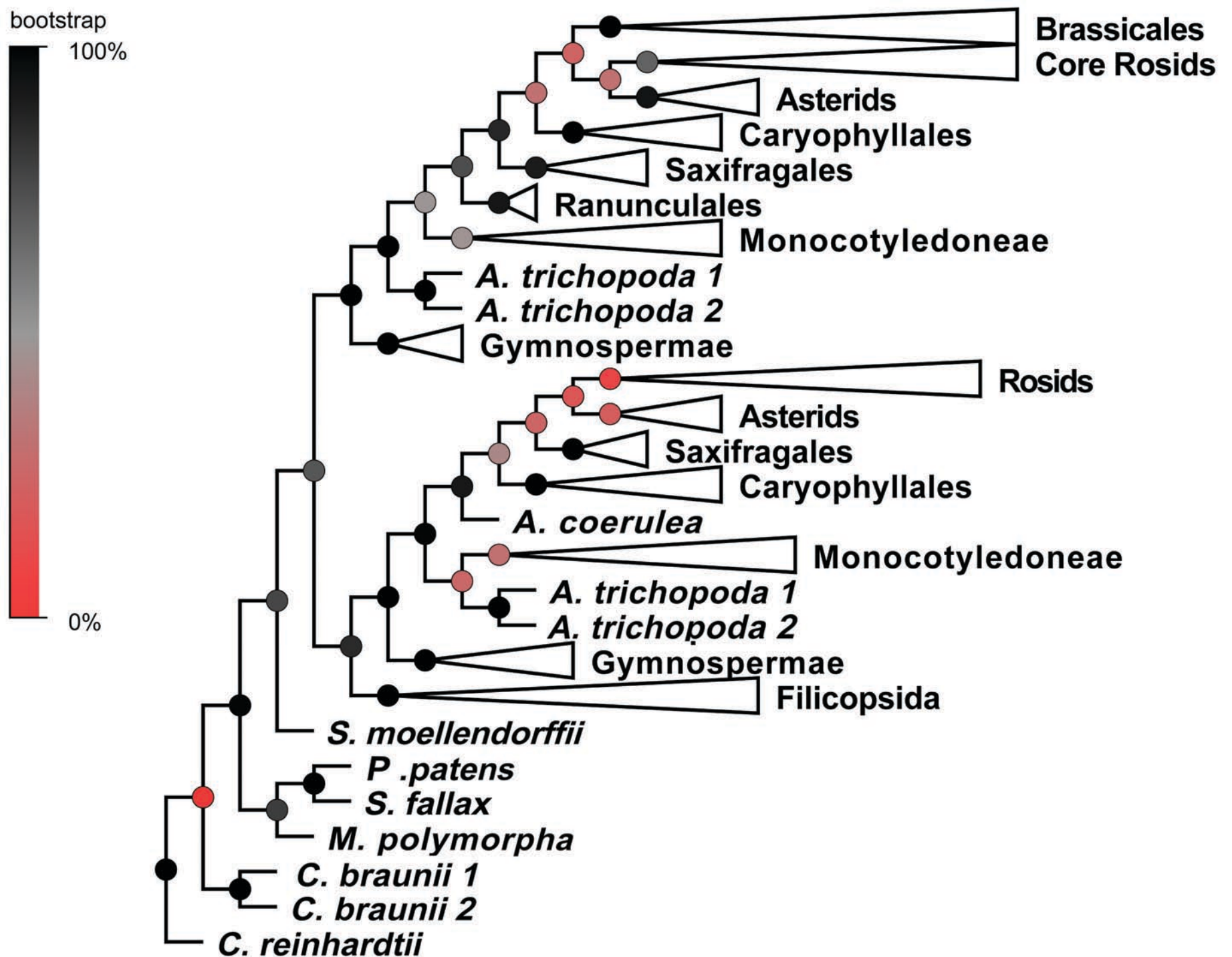

Supplement: Supplementary file 2 [file Image_2.pdf]

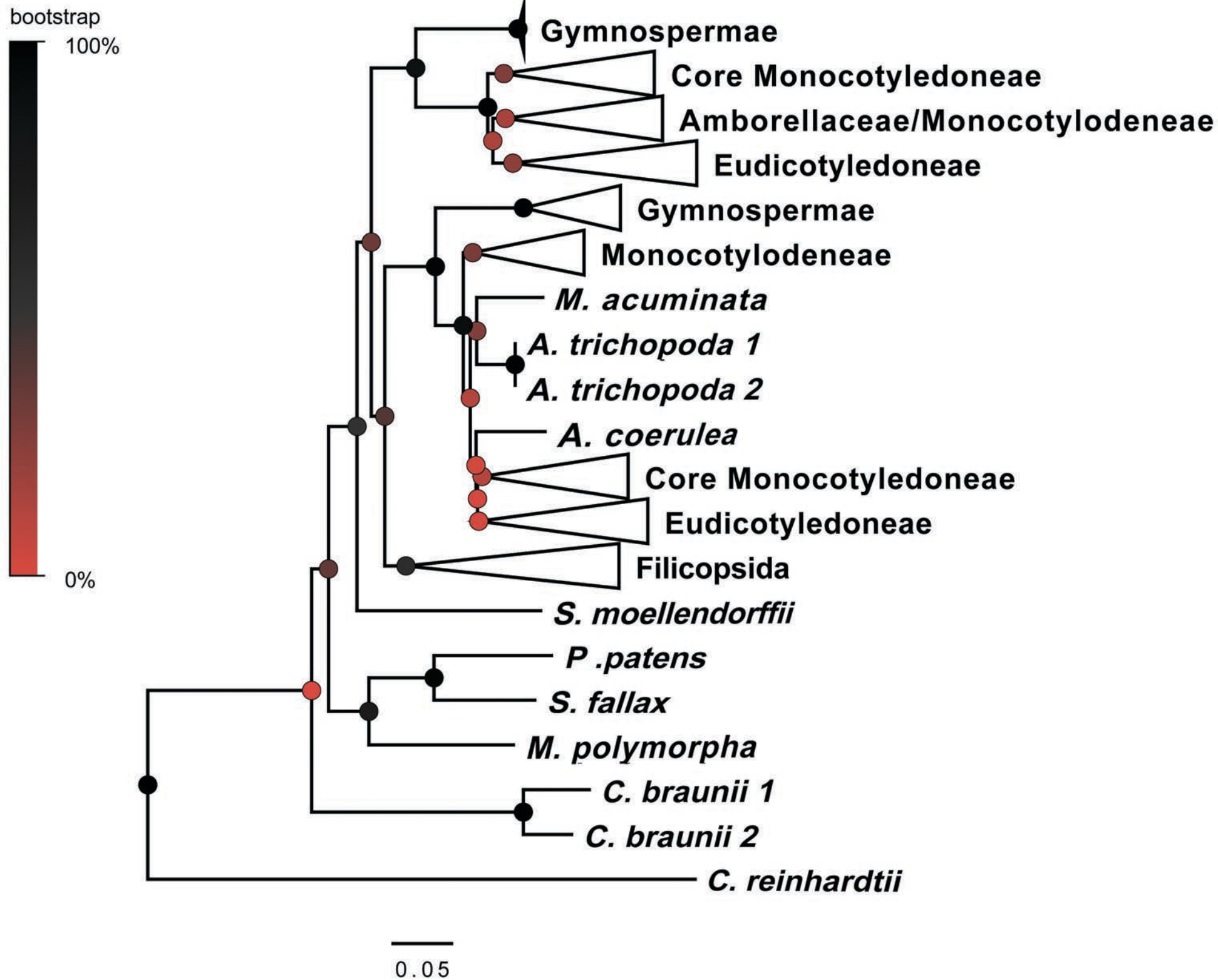

Supplement: Supplementary file 3 [file Image_3.pdf]

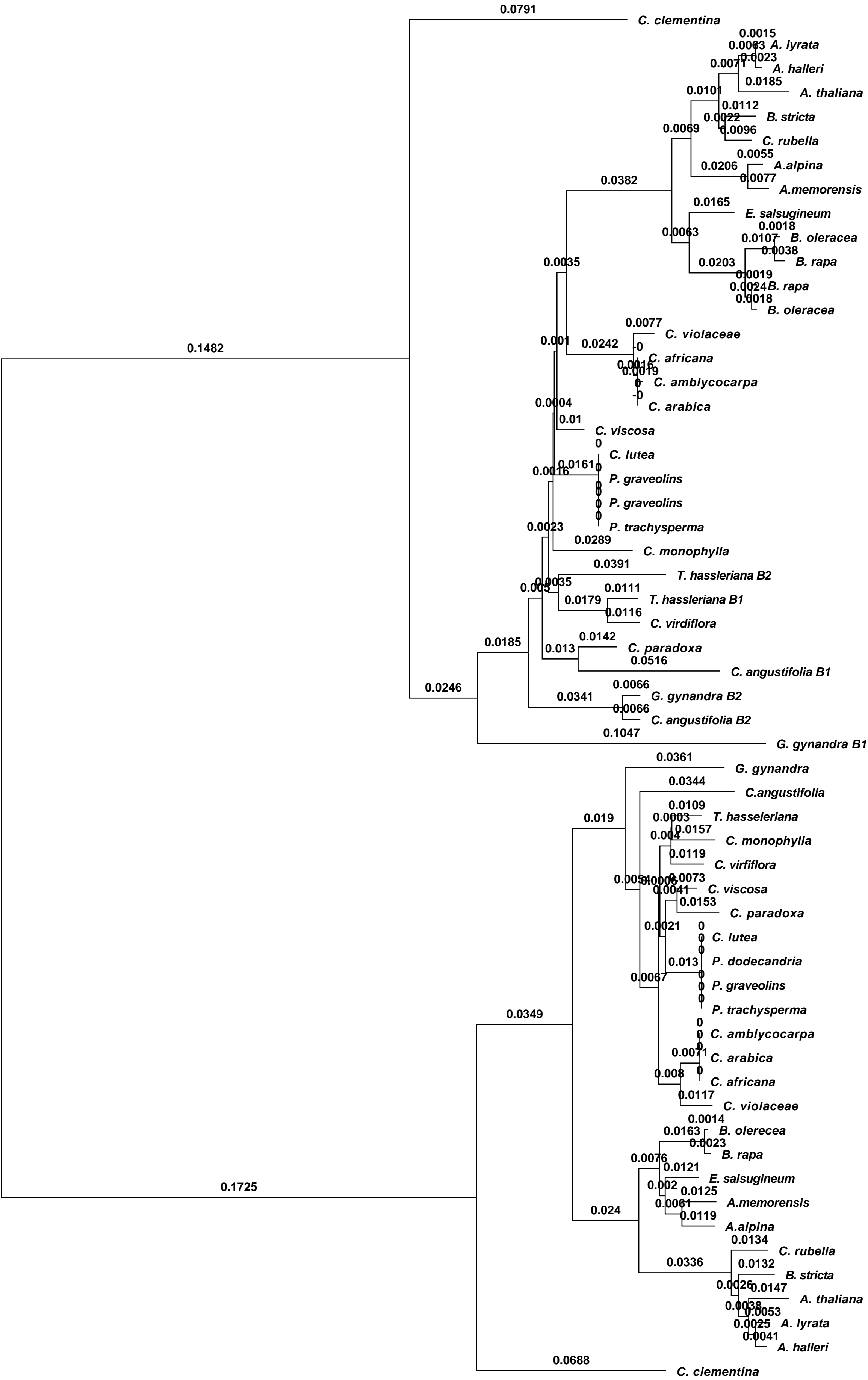

Supplement: Supplementary file 4 [file Image_4.pdf]

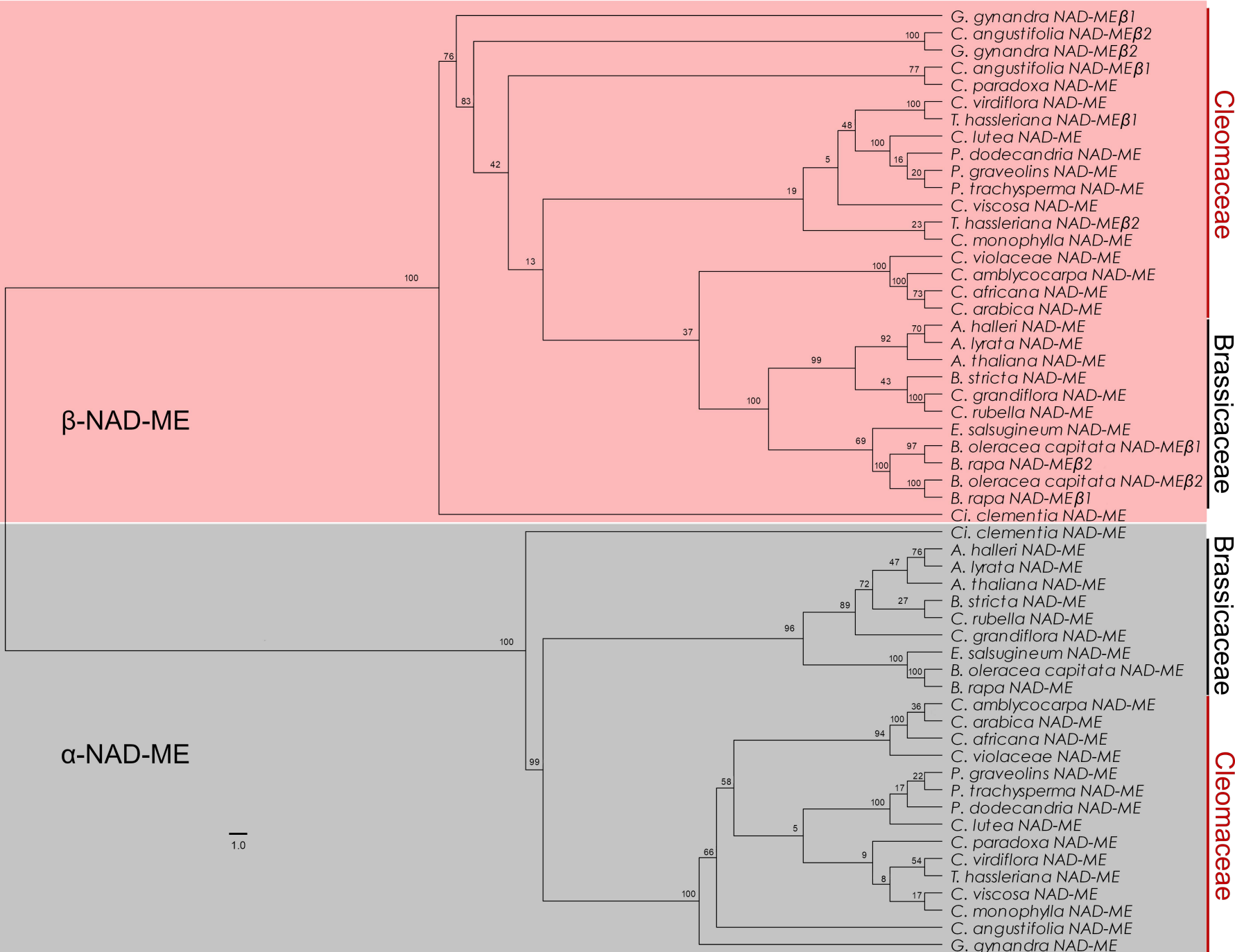

Supplement: Supplementary file 5 [file Image_5.pdf]

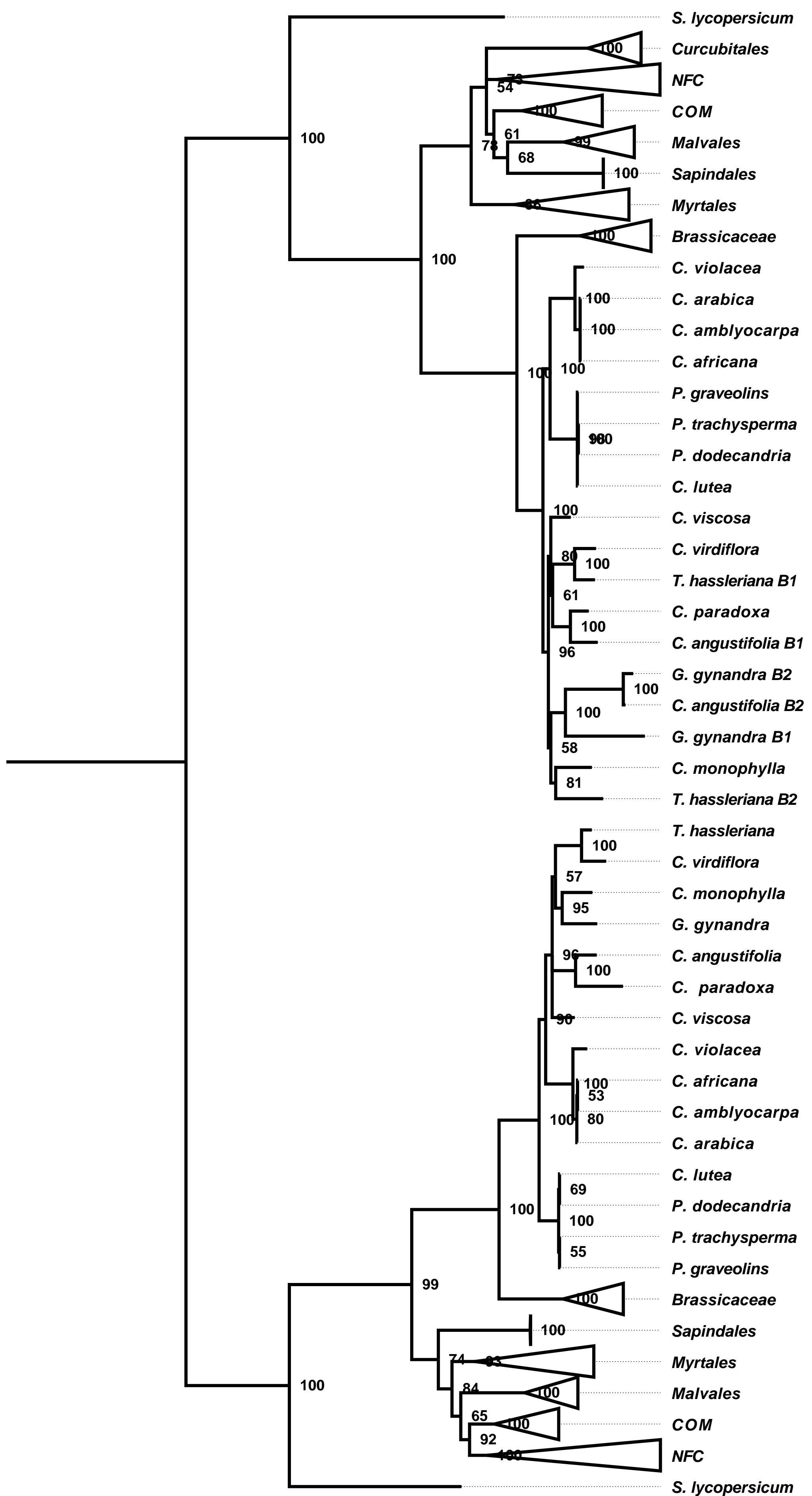

Supplement: Supplementary file 6 [file Image_6.pdf]

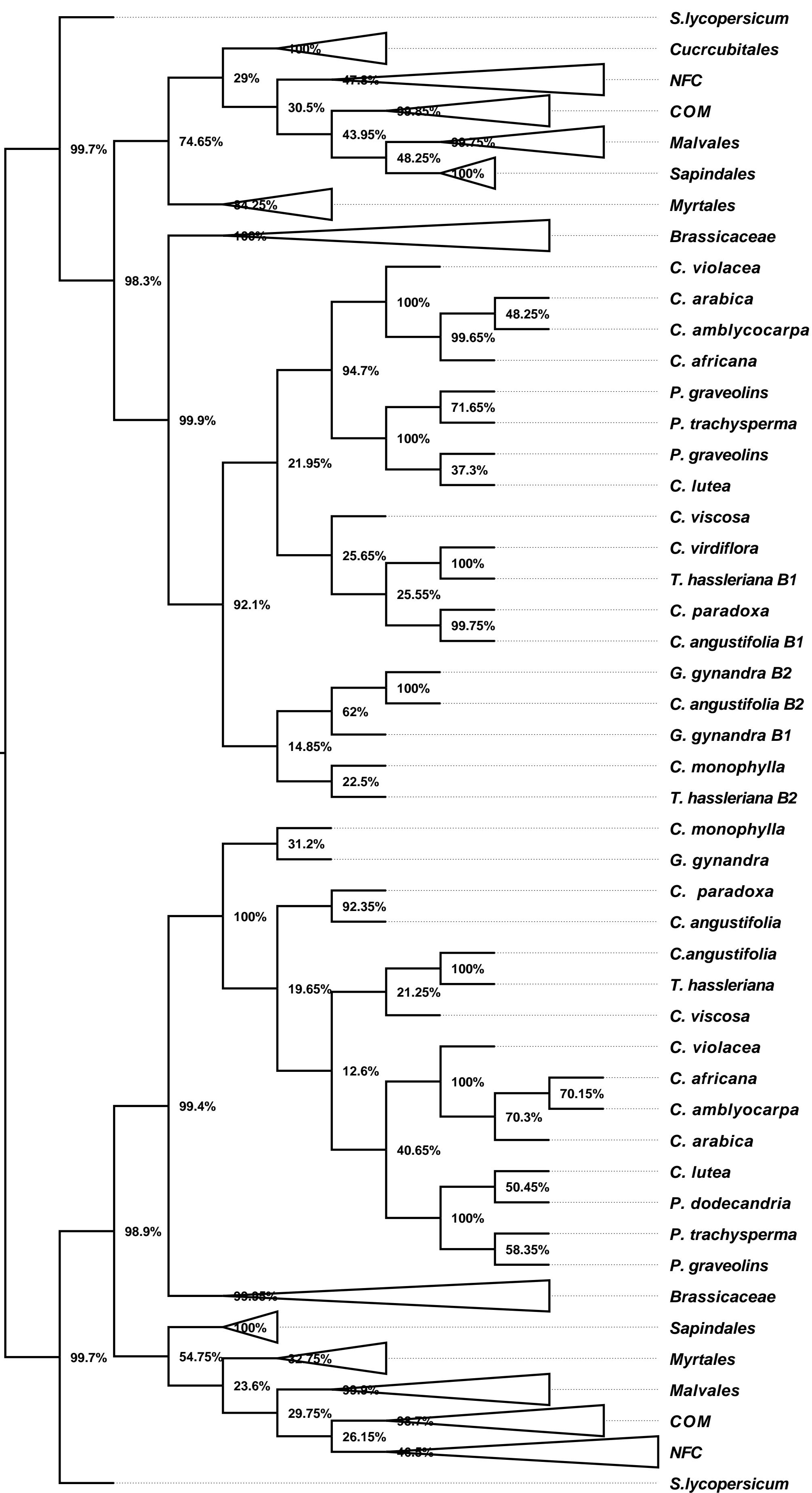

Supplement: Supplementary file 7 [file Image_7.pdf]
