# Supplementary material for: Independent Recruitment of Duplicated β-Subunit-Coding NAD-ME Genes Aided the Evolution of C4 Photosynthesis in Cleomaceae
Source: Front Plant Sci. 2020 Oct 6;11:572080. doi: 10.3389/fpls.2020.572080 (PMC7573226; doi:10.3389/fpls.2020.572080)
Supplement: Supplementary file 8 [file Data_Sheet_1.docx]

Supplementary Material

# Supplementary Data

**Supplementary Data 1.** Coding sequences-based BI tree fully resolved.

**Supplementary Data 2.** Coding sequences-based ML compressed tree.

**Supplementary Data 3.** Protein-based ML compressed tree.

**Supplementary Data 4.** Protein-based NJ tree of Brassicaceae and Cleomaceace fully resolved.

**Supplementary Data 5.** Protein-based ML tree of Brassicaceae and Cleomaceace fully resolved.

**Supplementary Data 6.** 3^rd^ position of codons BI tree fully resolved.

**Supplementary Data 7.** 3^rd^ position of codons ML tree fully resolved.

# Supplementary Figures and Tables

## Supplementary Figure

## Supplementary Figure 1. Alignment of β-NAD-ME protein sequences of Brassicales. Dots represent identical amino acids compared to the *G. gynandra* β1-NAD-ME (GgMEβ1) sequence. Amino acid positions are colored as follows: light blue, differentially substituted only in *G. gynandra* β1-NAD-ME; dark blue, differentially substituted only in *C. angustifolia* β1-NAD-ME (CaMEβ1); orange, differentially substituted in both sequences. In red letters, we highlighted the amino acid positions that were identified in the positive selection analysis following the numbering of the *G. gynandra* β1-NAD-ME sequence. Asterisks indicate substrate coordination sites (Chang and Tong, 2003). Boxes indicate protein domains (black, malate binding domain; magenta, NAD-binding domain).

**Supplementary Figure 2.** Alignment of α-NAD-ME protein sequences of Brassicales. Dots represent identical amino acids compared to the *G. gynandra* α-NAD-ME (GgMEα) sequence. Orange, differentially substituted amino acids in both *G. gynandra* and *C. angustifolia* (CaMEα) α-NAD-ME. Asterisks indicate substrate coordination sites (Chang and Tong, 2003). Boxes indicate protein domains (black, malate binding domain; magenta, NAD-binding domain).

**Supplementary Figure 1**

**
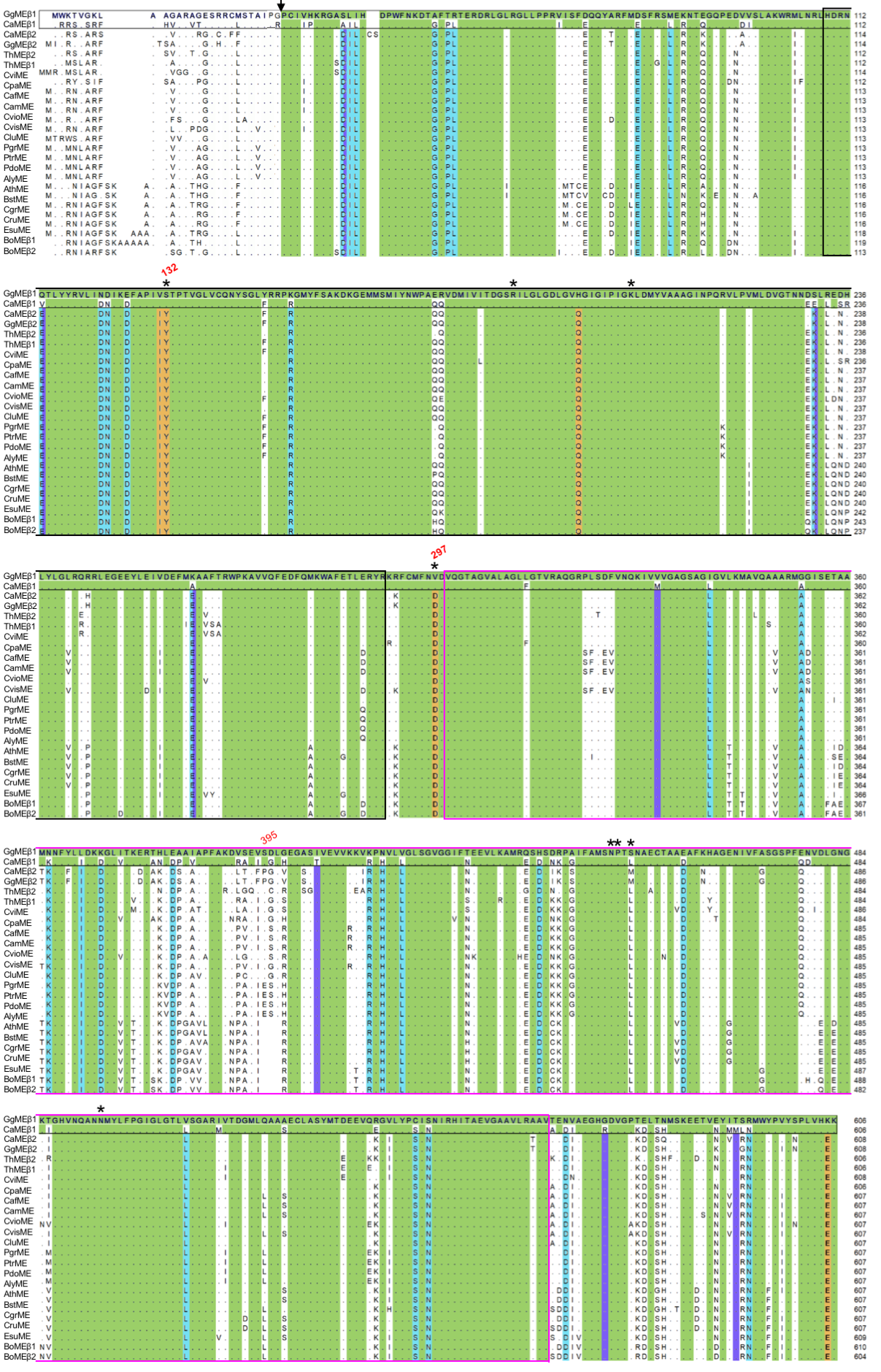
**

**Supplementary Figure 2**

**
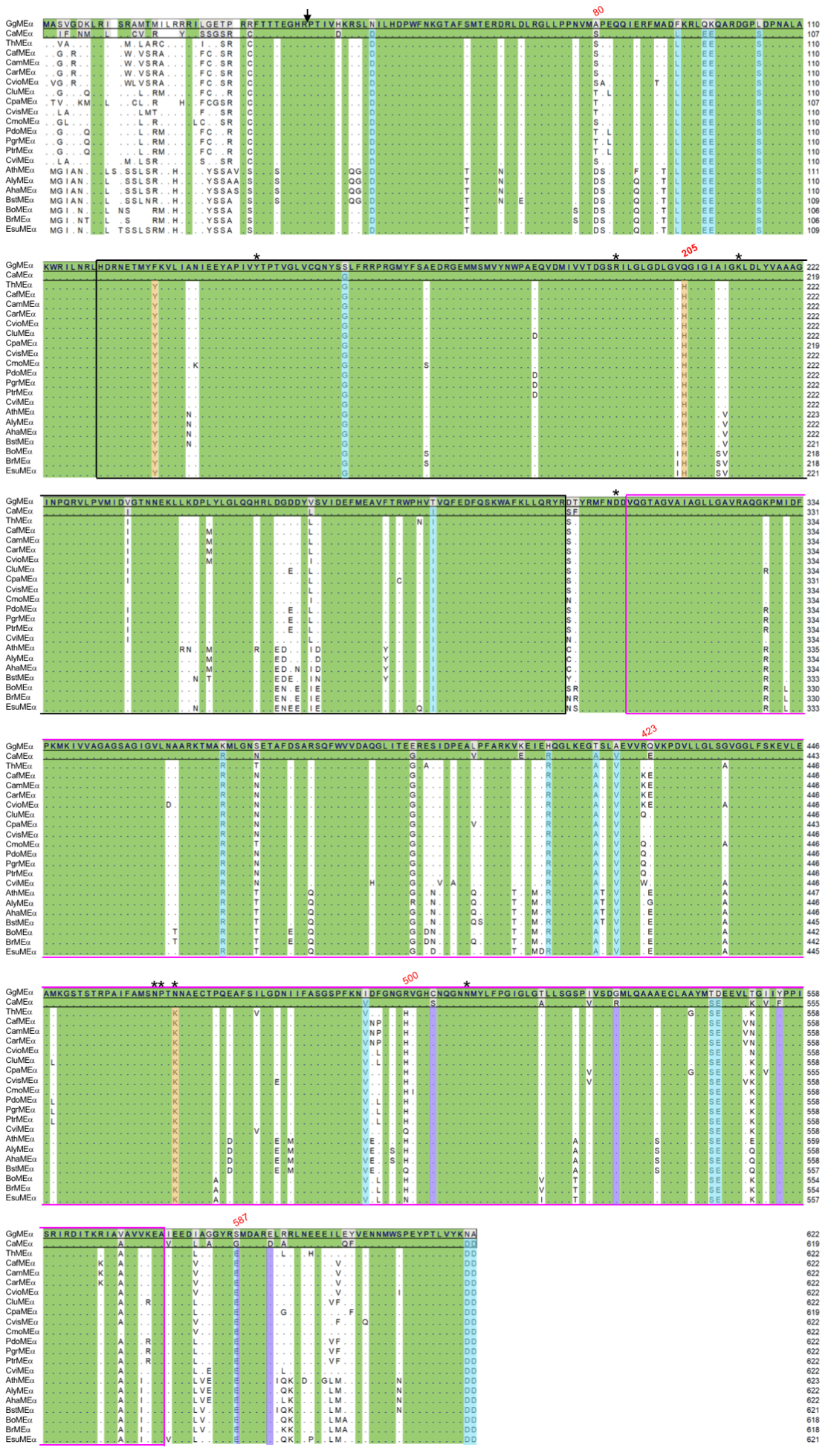
**

**2.2. Supplementary Tables**

**Supplementary Table 1.** Accession number of NAD-ME coding sequences used in this work.

A.alpina_CM002871.1

A.alpina_CM002874.1

A.coerulea_CDS_Aqcoe5G422700

A.coerulea_CDS_Aqcoe7G038700

A.coerulea_CDS_Aqcoe7G095300

A.edulis_comp18545_ME2

A.filliculoides_s0005.g009465

A.fokiensis_c20919_g1_RS48

A.formosae_c27927_g1_RS25

A.halleri_CDS_Araha.29463s0002

A.hypochondriacus_CDS_AH009241

A.hypochondriacus_CDS_AHYPO_1280

A.hypochondriacus_CDS_AHYPO_1281

A.lyrata_CDS_AL3G45400

A.lyrata_CDS_AL6G52860

A.memorensis_CABITT030000003.1

A.memorensis_CABITT030000006.1

A.officinalis_LOC109825172

A.officinalis_LOC109836847

A.pinnata_ssp_asiatica_c27412_g3

A.prostata_c12024

A.prostata_c31546

A.rosea_c22078

A.rosea_c3921

A.thaliana_CDS_AT2G13560

A.thaliana_CDS_AT4G00570

A.trichopoda_scaffold00002.109

A.trichopoda_scaffold00009.344

A.trichopoda_XM_006827678

A.trichopoda_XM_006838401

B.distachyon_CDS_Bradi1g27000

B.distachyon_CDS_Bradi3g30230

B.japonicum_c30430_g2_RS119

B.oleracea_CDS_Bol010756

B.oleracea_CDS_Bol011503

B.oleracea_CDS_Bol019325

B.rapa_CDS_Brara.C02902

B.rapa_CDS_Brara.I00034

B.rapa_CDS_Brara.I00951

B.stacei_CDS_Brast03G166900

B.stacei_CDS_Brast06G121100

B.stricta_CDS_Bostr.10064s0048

B.stricta_CDS_Bostr.13083s0038

C.africana_TRIN_DN11013c0g1i26

C.africana_TRIN_DN733c0g1i13

C.amblycocarpa_TRIN_DN17308c0g2i

C.amblycocarpa_TRIN_DN913c0g1i12

C.angustifolia_TRIN_DN18166-23437-30878-15844c0g1i (**β1**)

C.angustifolia_TRIN_DN31681c1g5i (**β1**)

C.angustifolia_TRIN_DN31760c0g1i (**α**)

C.arabica_TRIN_DN3739c0g1i1

C.arabica_TRIN_DN5939c0g1i12

C.braunii_G17566

C.braunii_G3306

C.capsularis_contig10546

C.capsularis_contig13345

C.clementina_CDS_Ciclev10019380m

C.clementina_CDS_Ciclev10024268m

C.lutea_TRIN_DN13865c0g1i14

C.lutea_TRIN_DN1911c0g1i14

C.melo_XP_008438266

C.melo_XP_008460179

C.monophylla_TRIN_DN12234c0g1i16

C.monophylla_TRIN_DN16339c0g1i12

C.moschata_LOC111432147

C.moschata_LOC111464523

C.paradoxa_TRIN_DN10143c0g1i26

C.paradoxa_TRIN_DN16613c0g2i66

C.quinoa_AUR62004314early-releas

C.quinoa_AUR62013799early-releas

C.quinoa_AUR62023359early-releas

C.quinoa_AUR62023443early-releas

C.reinhardtii_CDS_Cre06.g268750

C.rubella_CDS_Carubv10000504m

C.rubella_CDS_Carubv10013314m

C.sativa_LOC104737720

C.sativa_LOC104766630

C.sativa_LOC115706551

C.sativa_LOC115709970

C.sinensis_orange1.1g007456

C.sinensis_orange1.1g040403

C.violaceae_TRIN_DN3032c0g1i12

C.violaceae_TRIN_DN4958c2g1i12

C.virdiflora_TRIN_DN1304c0g2i15

C.virdiflora_TRIN_DN15251c1g1i13

C.viscosa_TRIN_DN2015c0g1i14

C.viscosa_TRIN_DN26986c0g1i16

D.antarctica_c9543_g1_RS43

D.carota_015776

D.carota_015950

D.catenatum_LOC110104377

D.catenatum_LOC110105405

D.zibethinus_LOC111294958

D.zibethinus_LOC111304697

E.diffusum_c37560_g1_i1_RS107

E.grandis_CDS_Eucgr.D02251

E.grandis_CDS_Eucgr.E01713

E.guineensis_LOC105042873

E.guineensis_LOC105053894

E.salsugineum_CDS_Thhalv10022600

E.salsugineum_CDS_Thhalv10028520

F.vesca_LOC101311414

F.vesca_XM_004306766

G.max_CDS_Glyma.03G014600

G.max_CDS_Glyma.03G102900

G.max_CDS_Glyma.07G074700

G.max_CDS_Glyma.09G262900

G.max_CDS_Glyma.18G229300

G.raimondii_CDS_Gorai.001G170700

G.raimondii_CDS_Gorai.007G097100

G.raimondii_CDS_Gorai.012G081300

G.gynandra _Ggy11557 (**α** )

G.gynandra_ Ggy18870 (**β1** )

G.gynandra_Ggy19628 (**β2** )

H.annuus_HanXRQChr01g0017481

H.annuus_HanXRQChr03g0093501

H.annuus_HanXRQChr04g0121671

H.annuus_HanXRQChr06g0168241

H.amboinensis_c18060_g1_RS19

H.brasiliensis_LOC110663918

H.umbratica_LOC110413175

H.umbratica_LOC110424986

J.curcas_LOC105643023

J.curcas_LOC105643271

K.fedtschenkoi_CDS_Kaladp0001s0130

K.fedtschenkoi_CDS_Kaladp0015s0134

K.fedtschenkoi_CDS_Kaladp0033s0124

K.fedtschenkoi_CDS_Kaladp0037s0467

K.fedtschenkoi_CDS_Kaladp0063s0037

K.fedtschenkoi_CDS_Kaladp0472s0027

K.laxiflora_CDS_Kalax.0025s0012

K.laxiflora_CDS_Kalax.0061s0109

K.laxiflora_CDS_Kalax.0146s0049

K.laxiflora_CDS_Kalax.0180s0037

K.laxiflora_CDS_Kalax.0321s0037

K.laxiflora_CDS_ Kalax.0197s0021

K.laxiflora_CDS_ Kalax.0229s0024

K.laxiflora_CDS_ Kalax.0400s0030

K.laxiflora_CDS_Kalax.0787s0012

L.matthewii_c25352_g1_RS70

M.acuminata_CDS_GSMUA_Achr1P0021

M.acuminata_CDS_GSMUA_Achr7P0683

M.charantia_LOC111016110

M.charantia_LOC111023088

M.domestica_CDS_MDP0000073044

M.domestica_CDS_MDP0000125870

M.domestica_CDS_MDP0000231868

M.esculenta_CDS_Manes.01G260100

M.esculenta_CDS_Manes.05G037900

M.esculenta_CDS_Manes.05G059200

M.guttatus_CDS_Migut.B01710

M.guttatus_CDS_Migut.N01711

M.polymorpha_CDS_Mapoly0080s0072

M.truncatula_CDS_Medtr7g076650

M.truncatula_CDS_Medtr8g009500

M.truncatula_CDS_Medtr8g032040

O.japonica_c33304_g1_RS38

O.sativa_CDS_LOC_Os07g31380

O.sativa_CDS_LOC_Os10g35960

O.thomaeum_CDS_Oropetium_09417A

O.thomaeum_CDS_Oropetium_11968A

O.thomaeum_CDS_Oropetium_13383A

P.aquilinum_c23616_g1_RS41

P.dactylifera_LOC103704635

P.dactylifera_LOC103712115

P.dodecandria_TRIN_DN10935c0g1i1

P.dodecandria_TRIN_DN41641c0g1i1

P.equestris_LOC110020669

P.equestris_LOC110024664

P.granatum_PG001100

P.granatum_scaffold130

P.graveolins_TRIN_DN10042c0g1i13

P.graveolins_TRIN_DN11309c0g1i14

P.hallii_CDS_Pahal.B04004

P.hallii_CDS_Pahal.I02893

P.makinoi_c53801_g1_RS111

P.miliaceum _PM02G10170

P.miliaceum _PM01G38550

P.miliaceum _PM04G25290

P.miliaceum _ PM03G07590

P.nudum_c28835_g1_RS21

P.patens_011G106000

P.persica_CDS_Ppe.2G114100

P.persica_CDS_Ppe.2G160000

P.pinaster_PPI00012428

P.pinaster_PPI00030324

P.pinaster_PPI00050340

P.pinaster_PPI00050663

P.simplex_c89446_g8_RS1

P.sitchensis_CDS_EF677094

P.sylvestris_PSY00011130

P.sylvestris_PSY00016680

P.sylvestris_PSY00017382

P.taeda_isotig36733

P.taeda_isotig41586

P.taeda_isotig42467

P.trachysperma_TRIN_DN29775c0g1i

P.trachysperma_TRINITY_DN35070_c

P.trichocarpa_CDS_Ptri.002G13530

P.trichocarpa_CDS_Ptri.002G15600

P.trichocarpa_CDS_Ptri.014G04370

P.trichocarpa_CDS_Ptri.014G07990

P.virgatum_2KG446000

P.virgatum_2NG499600

P.virgatum_8KG331900

P.virgatum_8NG228300

P.virgatum_9KG132400

P.virgatum_9NG251700

P.vittata_c24663_g1_RS36

P.vulgaris_CDS_Pvul.008G068700

P.vulgaris_CDS_Pvul.010G041000

P.vulgaris_CDS_Pvul.010G094700

R.argentea_LOC115678113

R.argentea_LOC115729088

R.communis_CDS_29709.m001210

R.communis_CDS_30174.m008988

R.sativus_LOC108827160

R.sativus_LOC108828697

S.bicolor_CDS_Sobic.001G201700

S.bicolor_CDS_Sobic.002G309400

S.cucullata_s0002.g000819

S.cucullata_s0185.g025020

S.fallax_CDS_Sphfalx0006s0328

S.italica_CDS_Seita.2G322000

S.italica_CDS_Seita.9G200600

S.lycopersicum_CDS_Solyc01g09420

S.lycopersicum_CDS_Solyc08g01386

S.moellendorffii_CDS_176472

S.oleracea_LOC110777831

S.oleracea_LOC110789122

S.polyrhiza_CDS_Spipo1G0035400

S.polyrhiza_CDS_Spipo5G0057800

S.tuberosum_PGSC0003DMG401000020

S.tuberosum_Z23023

S.viridis_CDS_Sevir.2G333400

S.viridis_CDS_Sevir.9G199800

T.cacao_Thecc1EG005393

T.cacao_Thecc1EG046943

T.pratense_mRNA10144

T.pratense_mRNA17976

T.pratense_mRNA23524

T. hassleriana _Th12536 (**α**)

T. hassleriana_ Th03046 (**β1**)

T. hassleriana_ Th09126 (**β2**)

V.vinifera_CDS_GSVIVT01018377001

V.vinifera_CDS_GSVIVT01026824001

W.prolifera_c45083_g1_RS128

Z.jujuba_LOC107421706

Z.jujuba_LOC107434740

Z.marina_CDS_Zosma133g00640

Z.marina_CDS_Zosma383g00030

Z.mays_CDS_GRMZM2G085747_P01

Z.mays_CDS_GRMZM2G406672_P01

**Supplementary Table 2.** Pairwise comparisons of site-class specific models through Likelihood-Ratio Test (LRT) and amino acids as evolving under positive selection detected by Bayes Empirical Bayes (BEB) in the α-NAD-MEs of Cleomaceae.

**Supplementary Table 3.** Pairwise comparisons of site-class specific models through Likelihood-Ratio Test (LRT) and amino acids as evolving under positive selection detected by Bayes Empirical Bayes (BEB) in the β-NAD-MEs of Cleomaceae.
